# Atomic Layer Deposition Processes: Versatile Platforms for Engineering ZnO‐Chitosan Biointerfaces

**DOI:** 10.1002/adhm.71329

**Published:** 2026-06-17

**Authors:** Mabel Moreno, Anjana Devi, David Zanders, Miryam Arredondo, Davide Mariotti, Ruairi McGlynn, Sindy Devis, Simón Guerrero, Eglantina Benavente, Matias Alegría, Yusser Olguin, Lorena Lobos‐Gonzalez, Kevin Guzmán, Elizabeth Rivas‐Yañez, Paula Solar, Valentin Cepus, Michael Krause, Luis Velásquez

**Affiliations:** ^1^ Instituto De Investigación Interdisciplinar En Ciencias Biomédicas SEK Facultad De Ciencias de La Salud Universidad SEK Metropolitan Chile; ^2^ Hochschule Merseburg − University of Applied Sciences Merseburg Germany; ^3^ Leibniz Institute for Solid State and Materials Research (IFW) Dresden Germany; ^4^ Faculty of Chemistry and Food Chemistry Dresden University of Technology (TUD) Dresden Germany; ^5^ Inorganic Materials Chemistry Ruhr‐University Bochum Bochum Germany; ^6^ Queen's University Belfast Belfast UK; ^7^ Department of Design Manufacturing & Engineering Management University of Strathclyde Glasgow UK; ^8^ Ulster University Belfast UK; ^9^ Facultad De Ciencias Químicas y Farmacéuticas Universidad De Chile Santiago Chile; ^10^ Departamento De Química Universidad Tecnológica Metropolitana Santiago Chile; ^11^ Programa Institucional de Fomento a la Investigación Desarrollo e Innovación (PIDi) Universidad Tecnológica Metropolitana Santiago Chile; ^12^ Departamento De Química y Medio Ambiente Universidad Técnica Federico Santa María Valparaíso Chile; ^13^ Centro científico y tecnológico de Valparaíso (CCTVal) Universidad Técnica Federico Santa María Valparaíso Chile; ^14^ Centro de Biotecnología Universidad Técnica Federico Santa María Valparaíso Chile; ^15^ Investigación en Dinámica Tumoral Mamaria (DiTMa Laboratorio De Comunicaciones Celulares Centro de Vesículas Extracelulares Metabolismo y Cancer (CEMC) Núcleo Interdisciplinario de Biología y Genética (NiBG) Instituto De Ciencias Biomédicas (ICBM) Facultad De Medicina Universidad De Chile Santiago Chile; ^16^ Facultad De Medicina Universidad De Atacama Copiapó Chile; ^17^ Departamento De Ciencias Biológicas y Químicas Facultad De Ciencias Universidad San Sebastián Santiago Chile; ^18^ Polymer Service GmbH Merseburg Merseburg Germany

**Keywords:** ALD techniques, angiogenesis, antiseptic ZnO coatings, biocompatibility, chitosan, in vivo integration

## Abstract

This study explores zinc‐functionalized chitosan (CS) for engineering bio‐multifunctional interfaces via three atomic‐scale techniques: vapor phase metalation (VPM), multiple pulsed vapor phase infiltration (MPI), and O2 plasma‐enhanced atomic layer deposition (PEALD). X‐ray photoelectron spectroscopy (XPS) analysis and scanning electron microscopy (SEM) with integrated energy‐dispersive X‐ray (EDX) elemental mapping confirmed homogeneous Zn distribution in all regimes, while AFM revealed a topographical transition from planarization in VPM (Rq = 5.6 nm) to high‐surface‐area nucleation in MPI (Rq = 123.9 nm). X‐ray diffraction (XRD) analysis demonstrated structural reconfiguration, with VPM reducing the hydrated phase crystallite size (7.4 to 4.6 nm) and MPI achieving the finest nanocrystallinity (1.83 nm). Notably, PEALD‐modified interfaces exhibited the highest interfacial energy (0.102 J/m^2^) and enhanced swelling. Physicochemical characterization showed the functionalization method dictates semiconductor properties, while biological assays revealed C2C12 cell proliferation comparable to the control, along with tailored antiseptic activity against *E. coli* and *H. pylori*. Significantly, in vivo subcutaneous implantation revealed that CS‐ZnO PEALD scaffolds act as immunomodulatory interfaces, promoting active angiogenesis and a balanced immune response with stable anti‐inflammatory IL‐10 levels and near‐basal pro‐inflammatory expression (IL‐6 = 0.5 pg/mL). These findings highlight the versatility of ALD‐based processes for next‐generation intelligent medical implants and bio‐integrated electronics.

## Graphical abstract

Vapour phase metalation (VPM), multiplied pulsed vapour phase infiltration (MPI), and O2 plasma‑enhanced atomic layer deposition (PEALD) tailor Zn–chitosan (Zn‑CS) films. PEALD improves wettability and biocompatibility. MPI enhances semiconductor behavior. Antiseptic selectivity: VPM → E. coli; MPI → H. pylori. PEALD boosts C2C12 viability and vascularization; Zn‑CS modulates IL‑6/IL‑10 balance.

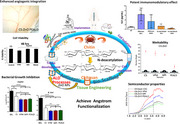

## 1 Introduction

Functionalized biopolymers represent a significant advancement in the development of bio‐multifunctional smart wearable sensors for medical devices [1, 2], tissue engineering [3, 4, 5], and optical Biological Micro‐Electro‐Mechanical Systems / Nano‐Electro‐Mechanical Systems (BioMEMS/NEMS) sensors [6, 7, 8]. These bioinspired platforms—leveraging hybrid biomaterials, swarm intelligence [9, 10], and quantum information/sensing [11, 12, 13, 14, 15, 16, 17]—are essential for sustainable technological progress and the advancement of conservation medicine across human, animal, and plant health. Such progress is driven by innovations in technological apparatuses, including high‐performance batteries, sensors, Organic Light‐Emitting Diode (OLEDs), and functional packaging [18, 19, 20, 21, 22]. Within this context, chitosan (CS) has emerged as a premier candidate for biomedical applications, owing to its exceptional biocompatibility and versatile biological profile [23, 24, 25].

**FIGURE 1:**
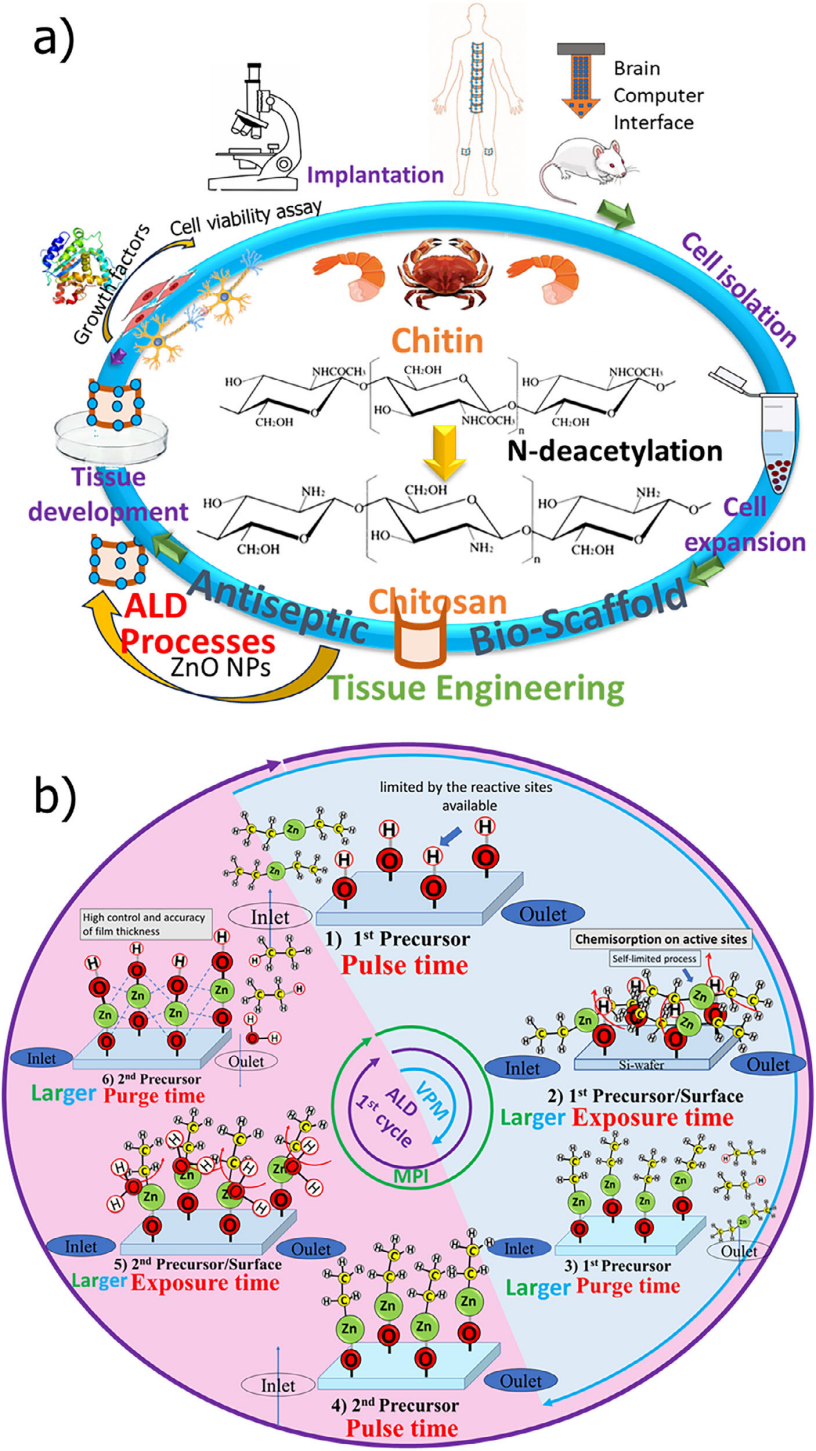
Schematic of chitosan scaffold‐based tissue engineering utilizing stem cells and atomic layer deposition (ALD) as a metallization functionalization tool. (a) Illustrative workflow of the extraction of chitin, its conversion to chitosan, and the development of functionalized bio‐scaffolds. (b) Scheme for synthesizing zinc oxide by ALD‐based techniques: (1) Diethylzinc (DEZ) pulse injection, (2) chemisorption of DEZ upon the Si wafer surface, (3) purge of unreacted DEZ and by‐products, (4) H_2_O pulse injection, (5) reaction of water with absorbed DEZ, and (6) purge of excess H_2_O and by‐products. Vapor‐phase metalation (VPM) subjects the substrate solely to the metal precursor for prolonged periods (the half‐cycle of ALD). In contrast, multiple pulsed vapor‐phase infiltration (MPI)—also known as sequential vapor‐infiltration or atomic layer infiltration—exposes the substrate to extended durations of both precursors to facilitate enhanced metal infiltration [67, 68, 69, 70, 71, 72, 73, 74, 75].

CS is a biodegradable polysaccharide derived from chitin, the second most abundant natural polymer after cellulose. It is produced commercially by deacetylation of chitin (Figure 1a), a structural element in the exoskeletons of crustaceans, insects, and invertebrates [26]. Deacetylation is often incomplete, so chitosan is defined as having a degree of deacetylation greater than 50% [27]. Chitosan's favorable properties, including biocompatibility, antiseptic activities, ability to cross the blood‐brain barrier (BBB) [28], mucoadhesiveness, and hydrogel‐forming capacity, have enabled its utilization in diverse applications such as dietary supplements (Glucosamine), joint support injections, pesticides, medical devices, drug delivery, and neural probes [23], and glial cell regeneration, the main responsible for maintaining homeostasis in neurons, forming myelin, provide support and protection [28].

While various polymers have been explored for mitigating glial scarring—a pivotal hindrance to neural interface devices [14, 23, 29, 30, 31, 32, 33, 34, 35, 36, 37, 38, 39, 40, 41, 42, 43, 44, 45, 46, 47]—chitosan stands out due to its superior biocompatibility and ease of functionalization. Notably, recent advancements have shown that chitosan, particularly when integrated with ZnO, exhibits unique fluorescent properties. This synergy makes it a premier candidate not only for structural scaffolds but also for bioimaging, disease diagnosis, and localized treatment [23, 48, 49, 50]

Chitosan's physicochemical properties, such as its ability to form polycationic species, good adhesion, film‐forming capability, high mechanical strength, and metal‐chelating capacity [46, 51], stem from its distinct molecular conformation, packing arrangement, and associated water molecules, as well as its hydroxyl and amino groups, rendering chitosan films a suitable material for anchoring antiseptic metals (e.g., ZnO, TiO_2_) [52, 53].

Scaffolds are platforms designed for implantation in the body to promote the growth of new tissues and repair damaged ones by providing the right signals to cells, thereby triggering biological mechanisms that encourage tissue expansion. The design of intelligent implants must consider eight key criteria [54]: storage capacity, mechanical properties, electrical stimulation, biocompatibility, angiogenesis, magnetic properties, biodegradability and absorptivity, and antiseptic properties.

Specifically, the scaffold must provide sufficient space for extracellular matrix regeneration and minimize diffusional constraints during in vitro culture [54, 55]. It must possess sufficient mechanical strength to avoid failure during implantation, support angiogenesis to satisfy blood supply demands, and exhibit magnetic properties to stimulate peripheral nerve regeneration [54, 56, 57]. Furthermore, the scaffold should be biocompatible and have an adjustable rate of biodegradability to provide temporary support that gradually transfers the mechanical load to the new tissue [54]. To further enhance the biological and functional performance of these scaffolds, the incorporation of bioactive elements such as zinc has emerged as a key strategy.

Zinc plays crucial roles in physiological and pathological processes, making it one of the most common and necessary components in brain function [58]. Additionally, zinc oxide is a direct wide bandgap semiconductor with a large exciton binding energy of 60 meV at room temperature [59]. Its unique properties, such as its use in transparent ZnO microprobe arrays for optogenetic neural manipulation, electroencephalogram and electrocorticogram electrodes [34, 45, 60], as well as its photocatalytic, electrical, optical, bactericidal, and fungicidal capabilities (which depend on particle size) [61, 62, 63], make it suitable for a wide range of scientific applications. The antiseptic features of ZnO arise from its ability to elevate the concentration of Zn^2+^ ions surrounding pathogens, which induces the generation of H_2_O_2_. This triggers irreversible bacterial membrane damage and excess production of nucleic acids and carbohydrates as a self‐defence mechanism in fungi, ultimately leading to cell death. Consequently, it is not surprising to find ZnO in various daily life products [64, 65]. Thus, using atomic‐scale controlled Zn metalation techniques could potentially boost the features of chitosan implants.

The classical Atomic Layer Deposition process [65, 66, 67, 68, 69, 70, 71, 72, 73, 74, 75, 76, 77, 78, 79, 80], also known as thermal ALD, involves the deposition of thin films through a series of self‐limiting, gas‐solid interface reactions. The process comprises the following steps: (1) The first precursor is injected into the ALD chamber in pulses for a specified time (e.g., diethylzinc). (2) The precursor chemisorbs onto the substrate surface, forming a monolayer during the exposure time. This leads to self‐termination, ensuring all available active sites on the substrate surface are loaded. (3) The excess precursor and byproducts are then purged from the chamber with an inert gas (e,g., Ar or N_2_). (4) The second precursor is injected in pulses to generate a monolayer of the final product. (5) The second precursor reacts with the first precursor absorbed on the substrate surface. (6) The excess second precursor and any byproducts are purged from the chamber with an inert gas. By repeating these cycles, the desired film thickness can be achieved (e.g., ZnO), typically around 0.1 nm per cycle, as shown in Figure 1b.

The thermal ALD process is highly adaptable and can be further improved through various techniques based on the same underlying principles. One such technique is Molecular Layer Deposition (MLD), which incorporates at least one organic molecule as a precursor [66]. Another approach is multiple pulsed vapor‐phase infiltration (MPI), also referred to as sequential vapor‐infiltration, sequential infiltration synthesis, atomic layer infiltration, or vapor Phase Infiltration (VIP). These techniques expose the substrate to extended durations of both ALD precursors to facilitate enhanced metal infiltration [67, 68, 69, 70, 71, 72, 73, 74, 75]. Additionally, vapor‐phase metalation (VPM) is a technique that subjects the substrate solely to the metal precursor for prolonged periods, a process also known as the half cycle, steps 1 to 3, as depicted in Figure 1b [67, 76, 77]. Plasma‐enhanced ALD and Radical Enhanced ALD processes, in which the substrate is exposed to plasma [78, 79] and radicals [80], respectively, are additional variations of the ALD technique.

After the pioneering work of George's group on Vapor Phase Infiltration [81], the functionalization of polymers by metal infiltration has received significant attention. This technique involves the deposition of Al_2_O_3_ through longer sequential pulses of TMA/H_2_O ALD cycles into polymers such as polystyrene [68]. Using a similar approach, it has been possible to infiltrate a range of materials, including spider dragline silks [82], cellulose [77], polyamide‐6 [70], polyester fibres [71], polyimide [73], conductive polymers [83], and carbonaceous materials [84], using ALD precursors like trimethylaluminum or diethylzinc [76]. This has enabled the selective metalation of methylmethacrylate blocks in block‐copolymers with phenylstyrene [85]. These processes have been successful in synthesizing metalated complexes and creating soft organic/inorganic hybrid materials with enhanced properties.

These processes have been successful in synthesizing metalated complexes and creating soft organic/inorganic hybrid materials with enhanced properties. Recent reports [67, 86, 87, 88] have pushed the boundaries of this technique, demonstrating its precision as a ‘nanosurgery’ tool for single molecular replacement in complex systems like enterobactin [86], as well as providing structural and biomedical insights into the metalation of polyamide‐6 [87]. This level of control was previously observed in the Zn functionalization of multi‐wall trititanate nanotubes [67], where VPM led to intercalation within the tube wall sheets, while MPI produced a hierarchical bi‐modal functionalization. Similarly, a structural metamorphosis has been recently achieved in the transformation of multilayered (NH_4_)_2_V_7_O_16_ squares into zinc vanadate and ZnO nanoparticles through precision metalation using the VPM technique [88]. Collectively, these studies demonstrate that the final architecture and properties are fundamentally determined by the presence of water—whether endogenous or exogenous—and the availability of reactive sites such as Ti‐OH or specific organic ligands.

In another study by Kääriäinen in 2011 [89], it was found that PE‐ALD could decrease the heat treatment of substrates near room temperature without destroying them, making it an attractive method for polymer functionalization. ZnO deposition has been widely reported using various zinc precursors and ALD processes. However, a comparative study of these processes on polymer films using the pyrophoric precursor DEZ has not been described. DEZ was selected due to its high vapor pressure, which facilitates the nucleation of ZnO at low deposition temperatures. There have been a few reported examples of chitosan functionalization using metal‐oxide Atomic Layer Deposition. For instance, Li et al. [90], demonstrated a SiO_x_ plasma‐enhanced atomic layer deposition (PEALD) transition coating between polylactic acid and chitosan, while Zhu [91] used ALD to deposit ZnO on chitosan‐modified carbon nanotubes. Hirvikorpi et al. [92], coated commercial polylactide films with a thin, non‐toxic polyelectrolyte multilayer film made from sodium alginate and chitosan, followed by a 25‐nm thick ALD Al_2_O_3_ layer. However, a comparative study of the metalation of chitosan using the aforementioned ALD approaches to improve polymer scaffold properties has not yet been reported. Moreover, none of the previous studies on chitosan–ZnO systems have reported bulk infiltration–induced structural refinement, electronic tuning, or in vivo angiogenic and immunomodulatory responses, which are central advances of the present work. The exceptional stability of hydrated chitosan may reflect strong interactions between chitosan and water molecules, providing an ideal environment for easily growing metal oxides such as ZnO and TiO_2_ using organometallic gases in an ALD chamber. This can potentially improve the scaffold and antiseptic properties. The present report focuses on studying the Zn functionalization of chitosan‐based membranes by carrying out VPM, MPI, and PE‐ALD processes using diethylzinc as precursors in a PE‐ALD reactor.

In this research, we focus on designing intelligent implants using chitosan (CS) and zinc‐functionalized chitosan (CS‐Zn) scaffolds. The study evaluates critical performance criteria, including swelling capacity, electrical and antiseptic properties, and biocompatibility. Surface topography and wetting characteristics were comprehensively analysed using Atomic Force Microscopy (AFM), contact angle (C.A.) measurements, and the determination of the apparent solid–liquid interfacial energy (∆G_SL_) to understand the hybrid interface's thermodynamic behavior. Electrical properties were assessed via the optical band gap, while antiseptic efficacy was tested against *Helicobacter pylori* and *Escherichia coli*. Biocompatibility was first evaluated in vitro using C2C12 myoblasts—cells essential for skeletal muscle regeneration—to explore the scaffolds' potential for treating sarcopenia, a condition affecting 10%–16% of the global elderly population [93]. Critically, the study culminates in an in vivo subcutaneous implantation model to validate tissue integration and angiogenic potential. This multifaceted approach sheds light on the development of next‐generation scaffolds for tissue engineering, sarcopenia treatment, and brain‐computer interface (BCI) devices.

## 2 Experimental

### 2.1 Film Synthesis

Chitosan films were prepared using the gel‐casting technique. A solution of 10 mL of 1% chitosan (75% deacetylated) in 0.2 M acetic acid is stirred for 48 h, then spread in a Petri dish and left to dry in the oven at 40°C for 72 h. The film is characterized by the solid‐state techniques already mentioned.

### 2.2 Thin Film Deposition and Vapor Infiltration

All vapor phase coating and infiltration experiments were performed in a custom‐built, hot‐wall stainless steel reactor chamber (20 cm x 20 cm x 20 cm) utilizing a top‐to‐bottom flow geometry for precursor and gas transport. The base pressure of the reactor system was 4×10^−5^ mbar, and operational pressures varied between 10^−2^ and 10^−3^ mbar. For the ZnO functionalization, diethyl zinc (DEZ) was employed. The respective precursors were fed to the reactor from stainless steel cartridges. Distilled water (H_2_O) was used as a co‐reactant and fed to the reactor from a separate line. Argon (AirLiquide, 99.995%) was employed as a purge gas, and its flow was adjusted to 15 sccm for all experiments. Chitosan samples were cut into 2‐inch pieces and functionalized as a film. During the experiments, DEZ was maintained at 30°C. For all VPM, MPI and ALD experiments, the chitosan samples were kept at 65°C. The different procedures for the functionalization of the chitosan samples are summarized in Table 1 and are based on prior ALD processes and VPM approaches previously conducted in this reactor [94, 95, 96].

**TABLE 1:** Samples name and their ALD synthesis method.

Samples ALD Method	Please provide Purge/pulse/exposure
VPM	5 cycles; DEZ Pulse: 3 s, DEZ Exposure: 40 s, DEZ Purge: 80 s
MPI	4 cycles; DEZ Pulse: 3 s, DEZ Exposure: 40 s, DEZ Purge: 80 s, H_2_O Pulse: 1 s, H_2_O Exposure: 40 s, H_2_O Purge: 120 s
PEALD	100 cycles; DEZ Pulse: 13 ms, DEZ Purge: 1 s, O_2_ Plasma Pulse: 150 ms, O_2_ Purge: 500 ms

### 2.3 Characterization

The products were characterized by XRD analysis (SIEMENS D‐5000, Cu‐Kα radiation) and Fourier transform infrared spectrometry (FT‐IR, Bruker Vector 22) at a spectral resolution of 4 cm^−1^ (KBr pellet). The diffuse reflectance UV–Vis spectra (Shimadzu UV–Vis model 2450 PC spectrophotometer with an integrating sphere) were recorded at room temperature in the 200–800 nm range at medium scan rates and with a 0.1 nm slit using barium sulphate as the reference. The reflectance measurements were converted to absorption spectra using the Kubelka–Munk function.

### 2.4 XPS

X‐ray photoelectron spectroscopy (XPS) was used in this study with the aim of analyzing the elemental composition and chemical bonding. The spectrometer was ThermoFisher ESCALAB XI+ instrument. The base pressure during spectra acquisition was better than 5×10^−7^ mbar achieved by an Edwards E2M28 rotary vane pump. The main background gas in the analysis chamber was argon at all points during the loading, pumping and measurements. The excitation source was a monochromated aluminum anode with an excitation energy of 1486.68 eV operated at approximately 15 kV and 15 mA. The work function of the spectrometer is determined by the software as 4.6 eV. With the selected scan parameters, the energy resolution was 0.1 eV for high‐resolution spectra and 1 eV for survey spectra. The C1s peak was set at 284.8 eV, a common approach for polymers [97, 98]. The size of the analysed sample area was 650 µm and takes the form of an elongated circle. For the XPS images, the detector employed a 150 µm square collection broken into 128 by 128 squares, each with a resolution of approximately 1.2 µm. The data was fit using ThermoFisher Avantage software, with peak assignment made using the internal library unless otherwise stated. The processed spectra were then copied to OriginPro for plotting.

### 2.5 SEM

The surface morphology and elemental distribution of the Zn‐functionalized chitosan samples (VPM, MPI, and PEALD) were investigated using a Tescan VEGA‐3 scanning electron microscope (SEM). To explore the changes in chemical composition and confirm the successful infiltration of zinc across the surface polymer matrix, the system was operated with an integrated energy‐dispersive X‐ray (EDX) spectroscopy unit. Micrographs were acquired at an accelerating voltage of 5 kV, which was optimized to prevent charging effects and beam‐induced damage to the organic matrix. While pristine chitosan was excluded from SEM analysis due to its highly insulating nature, the metalation and ZnO formation in the functionalized samples provided the requisite surface conductivity for high‐resolution imaging. EDX elemental mapping was further utilized to evaluate the homogeneity of the zinc species and to quantify the atomic percentages (at%) of C, O, N, and Zn, providing a direct correlation between the ALD process parameters and the resulting hybrid microstructure.

### 2.6 AFM

Surface topography was analysed using a Park Systems NX‐10 atomic force microscope operated in non‐contact mode. Large‐area scans were acquired over a 50 µm × 50 µm field with a resolution of 256 × 256 pixels. To visualize finer surface features, additional scans covering 5 µm × 5 µm at 512 × 512‐pixel resolution were evaluated. All measurements employed PPP‐NCHR cantilevers. Any tilt between the probe and the sample surface was corrected through line‐by‐line levelling.

### 2.7 Contact Angle

Contact angle (CA) measurements were carried out using a Pocket Goniometer P2 (Fibro System AB), and droplet images were processed with PocketGoniometer software version 1.7.4.0. Water was selected as the only probe liquid due to its biomedical relevance and its sensitivity to variations in surface polarity under aqueous conditions. It is widely used for characterizing chitosan‑based thin films and provides meaningful information on their wettability in physiologically relevant environments. The purpose of these measurements was to compare the relative hydrophilicity of the different CS–ZnO coatings rather than to determine full surface free energy, which would require multiple probe liquids. Experiments were performed at 25°C and 60 % relative humidity. Droplets were dispensed in steady‑state mode, and the resulting images were used to determine the contact angles.

For each sample type, 5–10 measurements were taken at distinct surface positions to ensure reproducibility. Reported values correspond to the mean of these independent measurements. To assess the energetic favorability of water interaction with the CS–ZnO surfaces, the apparent solid–liquid interfacial energy (−ΔG_SL_) was calculated using a modified Young–Dupré equation as described by Hurwitz et al. [99]: −ΔG_SL_ = (1+$\frac{\cos(\theta )}{r}$)×γ_*L*_ (eq. 1); where γ_*L*_ =  72.8 mN/m is the surface tension of water at 25°C, θ is the contact angle in degrees (converted to radians for cosine computation), and *r*  = 1+ (Rq/1000) is the roughness factor derived from AFM measurements.

### 2.8 In Vitro Cytotoxicity Characterisation

The C2C12 cells were subcultured on various samples of treated and untreated materials, including CS‐VPM, CS‐MPI, CS‐PEALD, and CS films, as well as control wells without any material, in a 24‐well plate. The C2C12 murine myoblast cell line was purchased from the European Collection of Authenticated Cell Cultures (ECACC, UK; Cat. No. 91031101). Each condition was performed in triplicate [100]. After three days of culture, cell growth was analysed through microscopic observation. Upon reaching confluence in both the control and sample wells, the culture was halted for analysis. The cells were then fixed using a methanol/paraformaldehyde protocol to preserve cellular structures. Fluorescence staining was subsequently performed using Hoechst dye to label the cell nuclei and Rhodamine/Phalloidin, a high‐affinity probe for F‐actin conjugated with the red‐orange, fluorescent dye Tetramethylrhodamine (TRITC), to visualize the cytoskeleton and cell cytoplasm [101].

### 2.9 Cell Viability Assay

The impact of surface treatments on cell viability was assessed using the WST‐1 assay, a widely utilized method for evaluating cellular metabolic activity. C2C12 cells were seeded at a density of 10^4^ cells per well in 96‐well plates. The WST‐1 colorimetric cell proliferation assays were conducted at 24 and 48‐h intervals following initial seeding to monitor the effects over time. For each assay, 10 µL of WST‐1 reagent was added to each well and incubated for 3 h at 37°C to allow optimal reagent interaction with metabolically active cells. The resulting color change, indicative of cell viability and proliferation, was measured at an absorbance of 450 nm using a microplate reader (BioTek ELx808). The absorbance values directly correlate with the number of viable cells in each well. Cell viability was calculated as a percentage of proliferation relative to untreated control cells, providing a comparative analysis of how different surface treatments influenced cellular growth. This approach ensures precise and reproducible results, facilitating the evaluation of the biocompatibility and efficacy of various surface modifications for biomedical applications. The WST‐1 assay's sensitivity and non‐radioactive nature make it particularly suitable for high‐throughput screening in biomaterials research [102, 103].

WST‐1 absorbance values were blank‐corrected and normalized to the untreated control at each time point, which was defined as 100% cell viability. Data are presented as mean ± SD from triplicate measurements (n  =  3). Statistical analysis was performed using one‐way ANOVA, considering surface treatment and incubation time as independent factors, followed by Tukey's post‐hoc test for multiple comparisons. Normality and homogeneity of variance were assessed using the Shapiro–Wilk and Levene tests, respectively. Outliers were evaluated by residual analysis and excluded only when attributable to technical or experimental errors. Statistical significance was set at p < 0.05. All analyses and graphs were generated using OriginPro software (OriginLab Corporation, Northampton, MA, USA).

### 2.10 Bacterial Growth Inhibition

Challenge culture of *Escherichia coli* K‐12 (ATCC 12435) and *Helicobacter pylori* 60190 (ATCC 49503). *E. coli* was grown in 5–10 mL 10% tryptic soy broth (Sigma‐Aldrich) for 24 h in a shaking incubator at 37°C. *H. pylori* was grown in Brucella broth (Sigma‐Aldrich) for 24 h in a shaking incubator at 37°C. Both bacteria were washed twice by centrifugation to replace the growth media with sterile water (2×2 mL tubes washed with 2×1 mL water) and diluted 1:1 (total 4 mL) to give a target range of 1.96 × 10^5^ CFU/mL after plating on agar.

The surfaces of modified membranes were prepared to 1 cm^2^, and, previous to the assay, were sterilized by UV light for 20 min per side. Then, each surface was inoculated with 100 µL from a tube containing 1 × 10^7^ CFU/mL of *E. coli* or *H. pylori* and maintained for 24 h at 37°C. The bacteria were recovered in PBS and seeded for colony counts by serial dilution over LB‐agar (Sigma‐Aldrich) for *E. coli* or Brucella Blood agar (Sigma‐Aldrich) for *H. pylori*. The colony count was performed after 48 h [104, 105].

### 2.11 In Vivo Assay

This study was performed according to the rules and standards established by the Bioethics Committee on Animal Research in the Faculty of Medicine, University of Chile (CBA 1169 FMUCH). Briefly, *four female BALB/c mice (*Mus musculus) aged 7–8 weeks** were maintained in specific pathogen‐free housing conditions, with controlled temperature and humidity, a 12 h light/dark cycle, and ad libitum access to food and water. Animals were subdivided randomly into different groups, and all were implanted using a minor surgical procedure. All animals were obtained from the institutional Animal Facility of the Faculty of Medicine, University of Chile.

Subcutaneous implantation of chitosan and chitosan–ZnO PEALD polymeric scaffolds was performed following a minimally invasive surgical approach, modified of the as previously described in our melanoma surgery models [106]. Animals were anaesthetized using isoflurane, and a CORE‐type implantation was carried out by local perforation without sutures. The polymer was introduced through the perforation site, and the small incision (maximum length of 10 mm) was closed by spontaneous approximation. Wound closure was allowed to proceed, and animals were euthanized at 96 and 168 h post‐implantation (n = 2 per condition and time point). Subcutaneous tissues were harvested and processed for anatomopathological and immunohistochemical analyses, following established protocols [106]. Systemic inflammatory responses were assessed by flow cytometry using the Mouse Inflammatory Cytokine Kit, quantifying circulating interleukins in plasma samples. Data acquisition was performed on a BD Cytometric Bead Array (CBA) Mouse Inflammation Kit measured in FACS Canto II flow cytometer at the Faculty of Sciences, University of Chile, under collaborative support Dra Maria Rosa Bono [106].

This in vivo experiment was designed as a pilot feasibility study, and the sample size (n = 2 per time point) was approved by the institutional ethics committee in accordance with the 3Rs principle (Replacement, Reduction, Refinement).

### 2.12 Ethical Approval

All in vivo experiments were performed in strict accordance with the guidelines and approval of the Institutional Committee for the Care and Use of Animals (CICUA) of the University of Chile. The study was conducted under Protocol CBA 1366 FMUCH, ensuring compliance with national and international ethical standards for animal welfare. All procedures were carried out under the professional supervision of the committee's designated authorities to ensure the ethical treatment and minimization of animal distress.

## 3 Results

### 3.1 X‐Ray Photoelectron Spectroscopy

**FIGURE 2:**
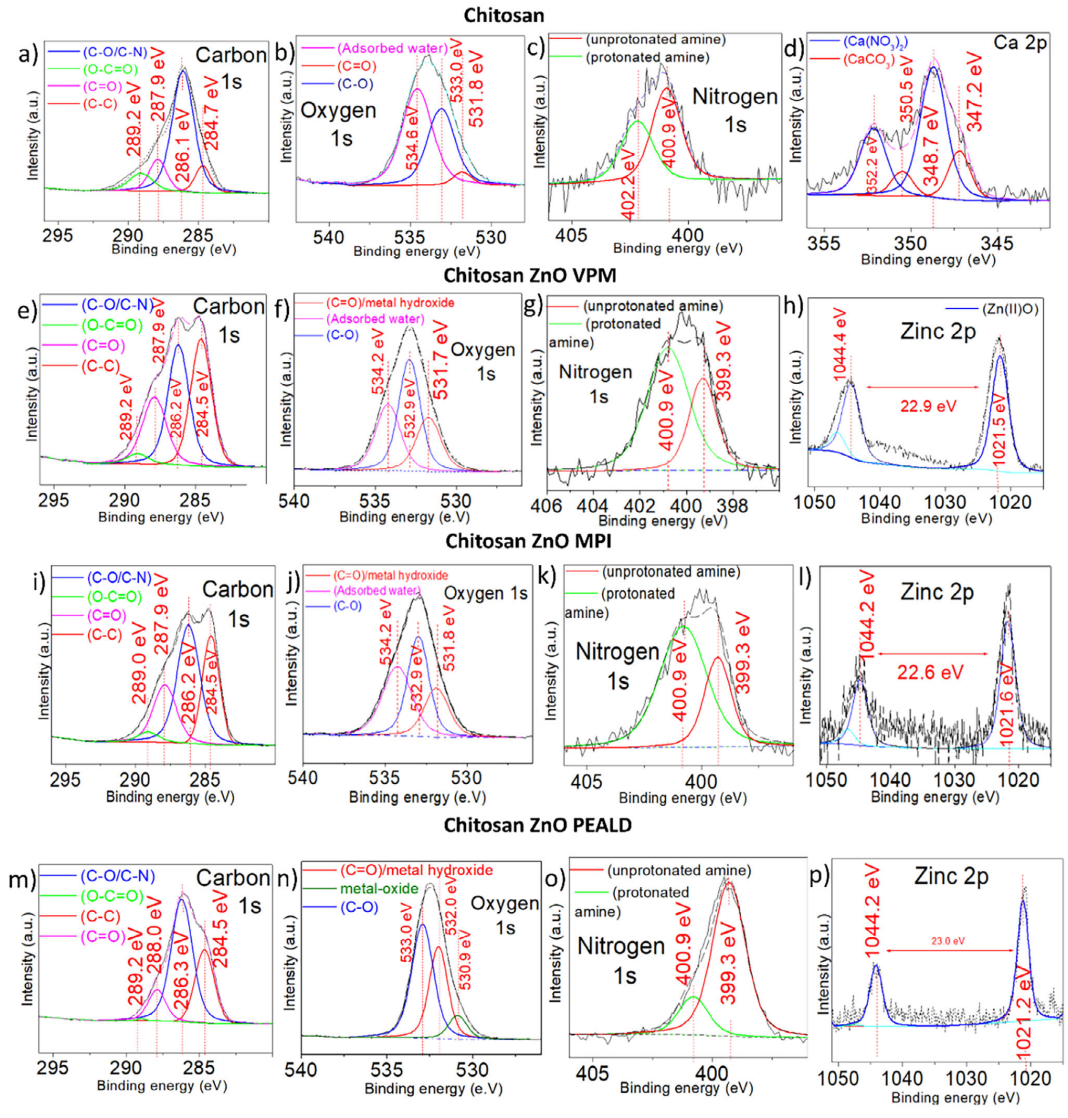
High‑resolution XPS spectra of the four samples: Chitosan (C 1s (a), O 1s (b), N 1s (c), Ca 2p (d)) previously reported and included here for comparison [109]; Chitosan–ZnO VPM (C 1s (e), O 1s (f), N 1s (g), Zn 2p (h)); Chitosan–ZnO MPI (C 1s (i), O 1s (j), N 1s (k), Zn 2p (l)); and Chitosan–ZnO PEALD (C 1s (m), O 1s (n), N 1s (o), Zn 2p (p)).

The high surface sensitivity of this method is a consequence of the inelastic mean free path of photoelectrons being limited to roughly 10 nm, with the bulk of the signal collected by the spectrometer arising between 4 and 6 nm [107]. Figure 2a‐d shows the high‐resolution spectra of pristine chitosan, where carbon is the primary element (78.5 at%), followed by oxygen (14.7 at%), nitrogen (3.9 at%), and calcium (2.8 at%). In the C 1s region (Figure 2a), the four components at 284.7, 286.1, 287.9, and 289.2 eV correspond to the expected polysaccharide backbone and absorbed acetic acid. The O 1s spectrum (Figure 2b) reveals major contributions from absorbed water (534.6 eV) and the polymer structure (533.0 eV), while the N 1s spectrum (Figure 2c) depicts unprotonated (400.9 eV) and minor protonated (402.2 eV) amine species. The Ca 2p peaks (Figure 2d) at 347.2 and 350.5 eV correspond to the CaCO_3_ and Ca^2+^ naturally present in the material. The transition to the VPM regime is illustrated in Figure 2e‐h, where the elemental composition shifts to 1.9 at% zinc, 27.3 at% oxygen, and 63.5 at% carbon. Compared to pristine chitosan, the C 1s spectrum (Figure 2e) exhibits a higher contribution from C‐C/C‐H bonds due to adsorbed reaction byproducts and a decrease in the ‐O‐C = O peak, suggesting Zn^2+^ induced CO_3_^2−^ hydrolysis [108]. Notably, the O 1s spectrum (Figure 2f) lacks a peak around 530 eV, confirming the absence of a stoichiometric metal‐oxide phase; instead, the higher contribution at 531.7 eV signals the presence of metal‐hydroxide. The consumption of the absorbed water peak in this panel underscores the high selectivity of DEZ for the structural water of the matrix, which is further supported by the N 1s region (Figure 2g), where an increase in the protonated amine peak reveals the synergism between N‐C = O, ‐NH_2_, and encapsulated water. The Zn 2p region (Figure 2h) confirms a 2+ valence state consistent with zinc carbonate and hydroxide species. In the MPI process, shown in Figure 2i‐l, a significantly lower zinc deposition is observed (0.4 at%), while carbon, oxygen, and nitrogen account for 70.2 at%, 23.3 at%, and 5.1 at%, respectively. The C 1s spectrum (Figure 2i) and the O 1s spectrum (Figure 2j) follow the VPM trend, lacking a metal‐oxide peak and favoring metal‐hydroxide signals at 531.8 eV. However, the slight alteration of the water peak in Figure 2j suggests that in MPI, the DEZ pulses react preferentially with the pulsed exogenous water, limiting infiltration compared to the VPM process. The N 1s spectrum (Figure 2k) maintains the signature of amine‐water synergism, and the Zn 2p profile (Figure 2l) remains consistent with zinc carbonate and hydroxide species. Finally, the PEALD regime presented in Figure 2m‐p reveals the most distinct chemical metamorphosis, yielding 0.4 at% zinc, 78.5 at% carbon, 14.7 at% oxygen, and 3.9 at% nitrogen. The C 1s spectrum (Figure 2m) shows a significant reduction in the ‐O‐C = O peak due to the decomposition effect of the O_2_ plasma. Most notably, the O 1s spectrum (Figure 2n) exhibits a new peak at 530.9 eV, which is the definitive fingerprint of ZnO lattice formation. The complete absence of an absorbed water peak in this panel indicates that the plasma‐enhanced process effectively dehydrates the matrix while promoting complete oxidation. This shift is mirrored in the N 1s region (Figure 2o), showing a substantial increase in unprotonated amines, suggesting that the plasma and DEZ preferentially strip protonated species. The Zn 2p spectrum (Figure 2p) reflects a complex mixture of zinc oxide, carbonate, and hydroxide species. All binding energy assignments are summarized in Table 2, with survey scans and Ca 2p regions for all samples provided in Figures S1–S3.

**TABLE 2:** The binding energies (eV±0.1) assigned to specific groups of CS and samples VPM, MPI and PEALD, namely: C 1s, O 1s, N 1s, Zn 2p and Ca 2p.

Element	CS	Sample VPM	Sample MPI	Sample PEALD	Assignment [110, 111]
C 1s	284.7	284.5	284.5	284.5	C‐C
	286.1	286.2	286.2	286.3	C‐O/C‐N
	287.9	287.9	287.9	288.0	C = O
	289.2	289.2	289.0	289.2	O‐C = O
N 1s	400.9	399.3	399.3	399.3	Unprotonated amine
	402.2	400.9	400.9	400.9	Protonated amine
O 1s	534.6	534.9	534.2	—	534.80/535.10 eV H_2_O
	533.0	532.9	532.9	533.0	Polysaccharide backbone (C‐O)
	531.8	531.7	531.8	532.0	(O–C‐O, N–C = O)/Zinc‐hydroxide
	—	—	—	530.9	Zinc‐oxide
Zn 2p	—	1021.5	1021.6	1021.2	Zn(II)
Ca 2p	347.2	347.1	347.2	347.1	CaCO_3_
	348.7	348.6	348.7	347.5	Ca(NO_3_)_2_
	350.6	350.8	350.4	350.2	CaCO_3_
	352.2	352.1	351.5	352.3	Ca(NO_3_)_2_

### 3.2 Elemental Distribution and Coating Uniformity

**FIGURE 3:**
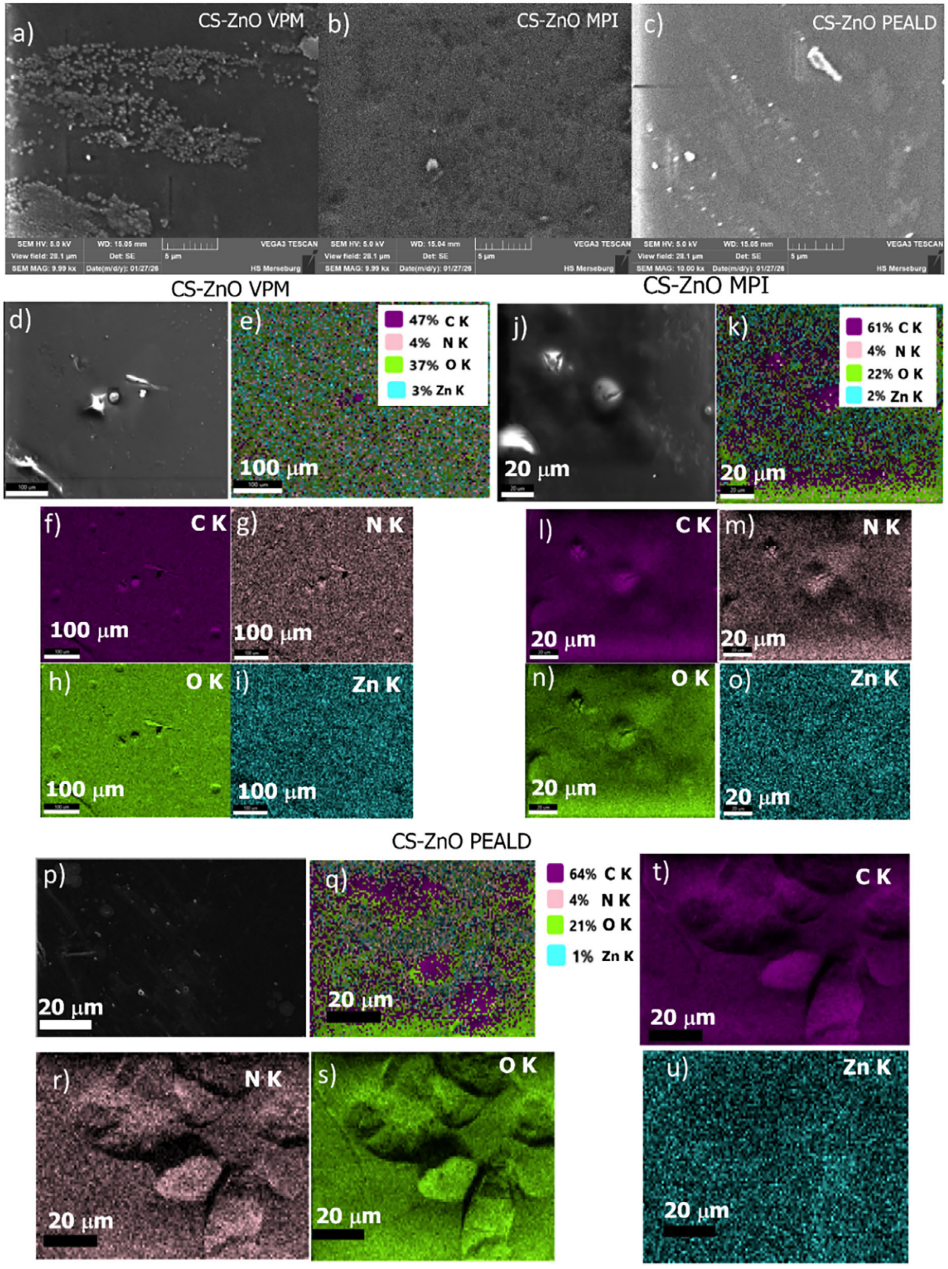
SEM micrographs and EDX elemental mapping of the CS‐ZnO samples. (a, c) Micrographs showing surface morphology with beam‐induced artefacts. (d‐u) Merged and individual EDX maps for C, N, O, and Zn, demonstrating homogeneous Zn‐infiltration throughout the chitosan matrix for CS‐VPM (d‐i), CS‐MPI (j‐o) and CS‐PEALD (p‐u).

Figure 3 presents the Scanning Electron Microscopy (SEM) micrographs and the corresponding Energy‑Dispersive X‑ray Spectroscopy (EDX) elemental maps for the CS‑ZnO scaffolds prepared via VPM, MPI, and PEALD. In the low‑magnification SEM images (a–c), all samples exhibit the characteristic sponge‑like morphology of chitosan. This intrinsic microstructure remains unchanged after Zn infiltration and ZnO deposition, indicating that none of the processes collapses or densifies the chitosan matrix. Across all three regimes, the EDX elemental maps show a homogeneous distribution of Zn and O throughout the scaffold, confirming that the Zn infiltration (VPM, MPI) and ZnO coating (PEALD) processes effectively penetrate the biopolymer network. The uniform Zn K (cyan) and O K (green) signals support the formation of continuous ultrathin layers rather than isolated aggregates. Chitosan is highly sensitive to electron‑beam exposure, and the surface roughness and irregularities observed in the SEM micrographs (d–u) arise from beam‑induced dehydration and local thermal degradation, rather than from the native topography of the scaffolds. The combined SEM‑EDX and XPS analyses demonstrate that the chitosan functional groups (–NH_2_ and –OH) act as high‑density nucleation and anchoring sites, enabling efficient Zn coordination and ZnO growth both at the surface and within the porous network. Based on the growth parameters (Table 1), the estimated coating thicknesses are <0.5 nm (VPM), ∼0.5 nm (MPI), and ∼10 nm (PEALD).

### 3.3 X‐Ray Diffraction and Optical Bandgap of Energy

**FIGURE 4:**
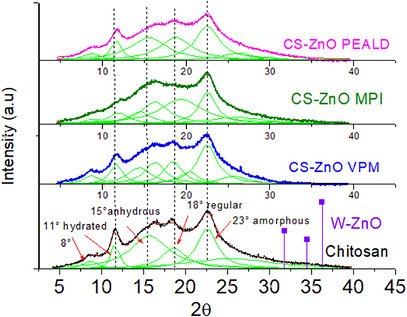
X‐ray diffraction patterns and structural deconvolution of CS–ZnO hybrid scaffolds. The diffractograms of VPM, MPI, and PEALD samples are contrasted with pristine CS and a Wurtzite‐ZnO reference. The experimental data (scatter) are overlaid with the cumulative multi‐peak deconvolution (solid lines) used to resolve the overlapping hydrated (11.6°), regular (18.6°), and amorphous phases of chitosan. Individual Lorentzian components illustrate the broadening and shift in full width at half maximum (FWHM) resulting from the different Zn‐functionalization processes, highlighting the structural refinement and dehydration effects across the varied ALD processing regimes.

Figure 4 displays the X‐ray diffraction patterns of pristine chitosan and its Zn‐functionalized derivatives (samples VPM, MPI, and PEALD). The specific 2θ reflections and their corresponding planes are summarized in Table 3, while the calculated crystallite sizes and the regular/hydrated area ratios are detailed in Table S1. The diffractogram of pristine CS exhibits five characteristic reflections. Based on previous studies [52, 53], the peaks at 8.5° and 11.6° are attributed to the hydrated crystalline structure of CS, resulting from the incorporation of water molecules within the crystal lattice. The reflection at 16.4° is assigned to an anhydrous crystalline network, while those at 18.6° and 22.5° correspond to the regular chitosan crystal lattice and the amorphous phase, respectively. Using pristine CS as a reference, sample VPM—prepared by pulsing solely DEZ solely—exhibits new reflections at 5.9° and 26.6°, signalling the formation of a distinct CS‐Zn coordination compound. In this regime, the crystallite size of the hydrated phase (11.6°) significantly decreases from 7.4 to 4.6 nm, while the regular phase (18.6°) slightly increases from 2.24 to 3.0 nm. This phenomenon confirms that, in the absence of an exogenous second precursor, the DEZ molecules preferentially consume the nucleated water sources within the CS lattice to drive the metalation. In contrast, the MPI and PEALD processes trigger a more profound structural reconfiguration. For the MPI sample, the crystallite size of the hydrated phase drops further to 2.8 nm, and the regular phase reduces to 1.83 nm. This widespread reduction in crystallite size across all phases suggests that the multiple pulses of exogenous water in MPI promote a high density of nucleation sites, preventing the growth of large crystal domains—a finding that correlates with the high surface roughness (Rq) observed in AFM. Finally, the introduction of a second precursor in MPI and PEALD leads to an optimized regular‐to‐hydrated area ratio of 2.1, notably higher than the 1.7 observed in the VPM regime. This suggests that while VPM primarily targets internal water for metalation, the MPI and PEALD processes (utilizing H_2_O and O_2_ plasma, respectively) facilitate a more balanced reorganization of the crystalline network. The R^2^ deconvolution values for all XRD patterns exceeded 0.994 using Lorentzian functions, ensuring the reliability of the peak fitting. These alterations underscore the synergistic interactions between Zn‐functionalization and the CS lattice, where the deep structural metamorphosis observed in two‐precursor processes contrasts with the selective metalation of hydrated regions under VPM conditions. These alterations not only underscore the synergistic interactions between N–C = O, ‐NH_2_, and encapsulated water within the CS structure but also emphasize the inherent resilience of this biopolymer. This resilience offers insights into the adaptability of crustaceans throughout Earth's history, particularly during periods of significant environmental change, such as volcanic eruptions.

**TABLE 3:** XRD reflections in 2θ and their corresponding planes assigned for CS film, samples VPM, MPI and PEALD.

Hydrated CS RT‐40°C (plane)* [52] In 2 θ	Anhydrous CS 60–80°C (plane)*[ 52 *]*	Commercial pure powder CS High molecular weight [53]	Current CS film	Sample VPM	Sample MPI	Sample PEALD
8.5°		8.3° sh *(200)*	8.5°	8.7	9.1	8.8
11.8° *(110)*		11.6° *(020)*	11.6°	11.7	12	11.7
16.6° *(020)*	15.4°*(110)*	16.2°	16.4°	16.4	16.3 (w.sh)	15.7
18.4°		18.4°*(220)*	18.4°	18.4	18.5	18.7
22.9°	21.3° *(020)*	23.1° *(220 and 202)*	22.7°	22.7	22.6	22.5
	23.9°*(120)*					

**FIGURE 5:**
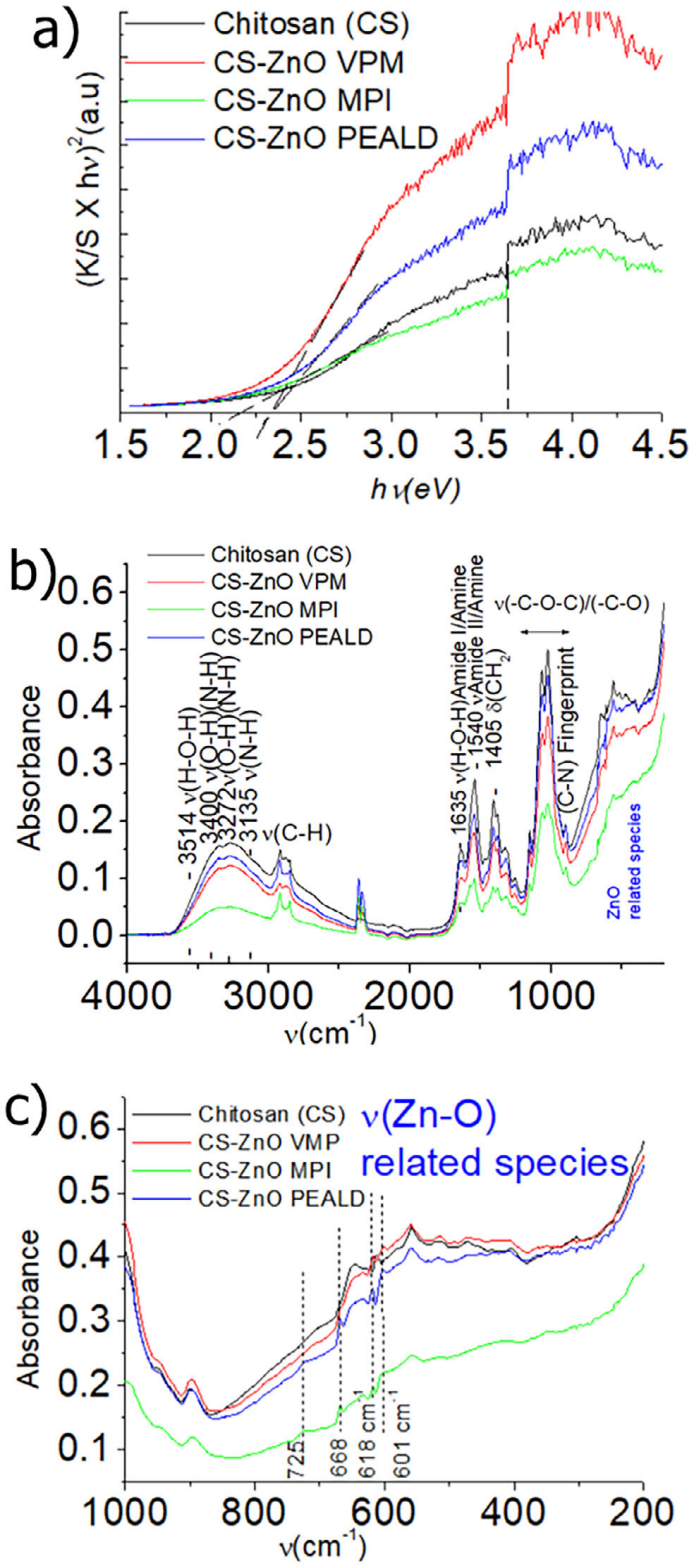
(a) Plot (K/S × hν)^2^ against hν showing the linear fitting to the main linear segment of the curve considering a direct band gap transition, according to the Tauc equation obtained by reflectance diffuse spectra (after Kubelka–Mulk transformation) [112] of samples VPM, MPI and PEALD and Chitosan. (b,c) ATR‐FTIR spectra of Chitosan and its DEZ functionalized films; samples VMP, MPI and PEALD.

Figure 5a illustrates the Tauc plots – (K/S × hν)^2^ versus hν – derived from the reflectance diffuse spectra (after Kubelka–Mulk transformation) [112] of pristine chitosan, sample VPM, sample MPI, and sample PEALD. These plots, based on the Tauc equation, allow for determining the optical band gap, assuming a direct band gap transition. A clear redshift of the absorption band edge is observed in all modified samples compared to bulk w‐ZnO (Eg: 3.21 eV), with all values summarized in Table 4. This shift indicates a modification in the electronic band structure of the ZnO upon complexation with chitosan. The extent of the redshift follows the order: VPM > PEALD > MPI, similar to trends reported for Ca‐doped ZnO [113] and other ZnO arrays, where broadband gap windows ranging from 2.8 eV to 4.2 eV have been observed [62, 67, 114, 115, 116, 117, 118].

**TABLE 4:** Optical bandgap energy (Eg) values for pristine chitosan, VPM, MPI, and PEALD samples compared to bulk w‐ZnO.

Sample	eV
w‐ZnO	3.21 ± 0.03 [115]
Sample VPM	2.25
Sample MPI	2.16
Sample PEALD	2.31

**FIGURE 6:**
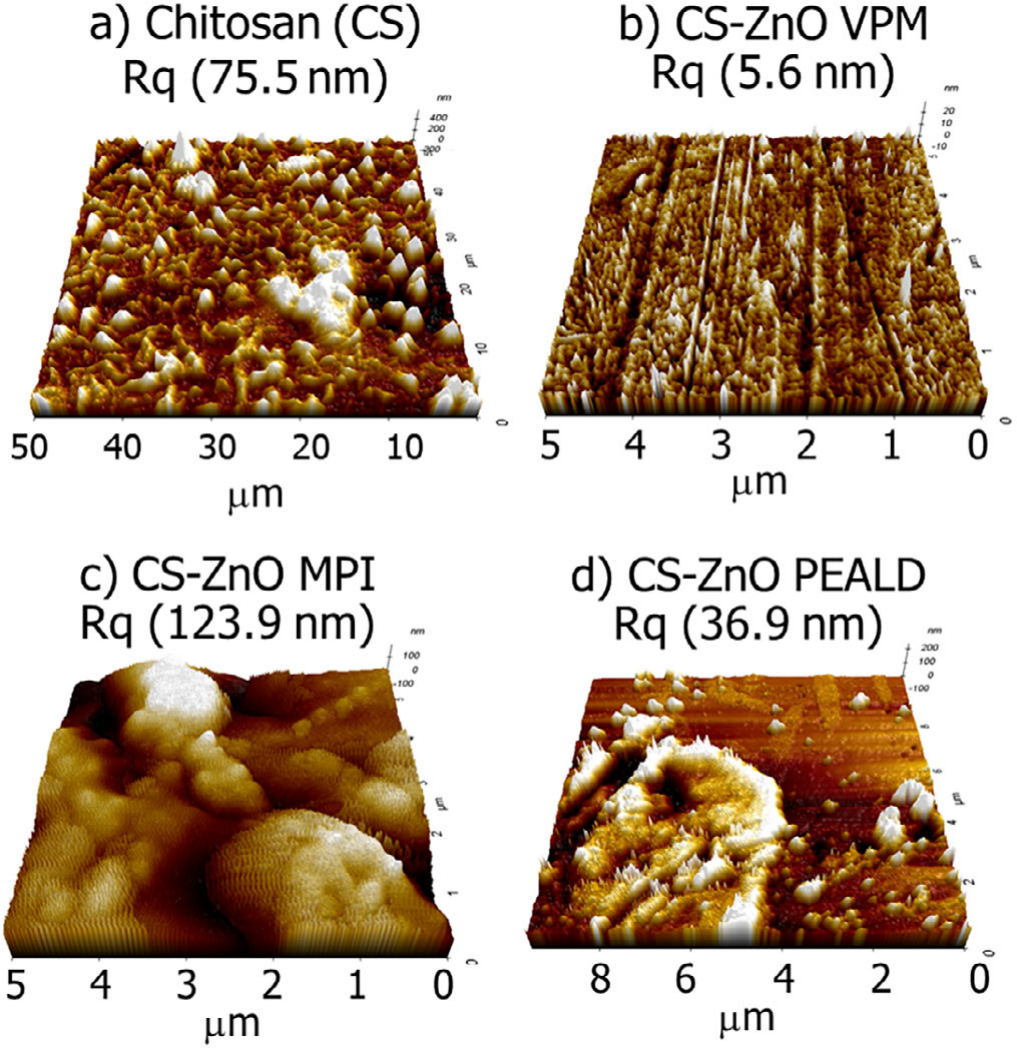
AFM surface 3D topography of chitosan‐coated ZnO layers synthesized via three distinct ALD methods: (a) Chitosan (CS), CS‐ZnO‐VPM (b), CS‐ZnO‐MPI (c), and CS‐ZnO‐PEALD (d).

Figure 6b, c show the ATR‐FTIR (Attenuated Total Reflectance‐Fourier Transform Infrared) spectroscopy results, which, with a shallow penetration depth of 0.5 to 2 µm, are an excellent tool for analyzing thin films and surface layers. This technique is particularly effective for characterising modifications in polymer films induced by the DEZ‐VPM, MPI, and PEALD processes. Figures S4–S6 show the deconvolution of the ATR‑FTIR spectra for the VPM, MPI, and PEALD samples in the 1000–2000 and 2000–4000 cm^−^^1^ regions, providing resolved vibrational components that support the spectral assignments described here. The characteristic absorption bands and their corresponding vibrational assignments for the pristine chitosan and functionalized films are summarized in Table 5. As confirmed by X‐ray Photoelectron Spectroscopy (XPS), these processes alter the chitosan (CS) films, which initially comprise 70% chitosan and 30% chitin and display characteristic peaks in the ATR‐FTIR spectrum (Figure 6b). Upon modification with diethylzinc (DEZ) using atomic layer deposition (ALD), notable spectral changes include a decrease in the O‐H stretching band at 3544 cm^−^^1^ (related to encapsulated water) and a decreased and broader peak at 1633 cm^−^^1^ (due to bending vibrations of encapsulated water and overlapping with amide and amine bands). The peak at 1470 cm^−^^1^ almost disappears, reflecting a significant reduction or changes in amide groups due to interactions with zinc species [116, 117]. Additionally, the peak at 1410 cm^−^^1^ decreases in intensity, possibly due to interactions affecting carbonate‐related groups. The increased intensity of the peak at 1566 cm^−^^1^ suggests enhanced N‐H stretching or bending vibrations, observed in XPS spectra. The emergence of peaks at 601, 618, and 725 cm^−^^1^ is indicative of Zn‐O stretching in zinc hydroxide (Figure 6c), as observed in XPS. On the other side, the MPI sample involves the pulsing of DEZ and water onto the CS film, leading to notable spectral changes. ATR‐FTIR analysis reveals a significant decrease in the O‐H stretching band (3000–3700 cm^−^^1^) due to reduced encapsulated water, and decreased peaks at 1636, 1547, 1375, and 1410 cm^−^^1^, reflecting changes in amide and carbonate groups. The peak at 1584 cm^−^^1^ increases, suggesting enhanced N‐H stretching or bending vibrations due to the external source of water in the sample. New peaks at 602, 619, and 725 cm^−^^1^, along with a shoulder at 991 cm^−^^1^, indicate Zn‐O stretching in zinc hydroxide. The ATR‐FTIR spectra of the DEZ‐PEALD sample exhibit several distinctive features, indicating significant interactions between DEZ and the chitosan matrix under O_2_ plasma‐enhanced ALD conditions. Notable peaks at 601, 619, 668, and 725 cm^−^^1^ correspond to Zn‐O stretching in zinc hydroxide and related species. A shoulder at 992 cm^−^^1^ suggests additional interactions within the modified chitosan film. The peak at 1022 cm^−^^1^ decreases, while the peak at 1062 cm^−^^1^ increases, reflecting changes in C‐O stretching vibrations related to carbonate groups (Table 5). Peaks at 1317 and 1410 cm^−^^1^ decrease in intensity, likely due to interactions affecting carbonate‐related groups, while the peak at 1472 cm^−^^1^ almost disappears, indicating changes in amide groups. The band between 1481 and 1605 cm^−^^1^, containing peaks at 1543 and 1576 cm^−^^1^, is broader than in the pristine chitosan film, suggesting enhanced N‐H stretching or bending vibrations. The peak at 1632 cm^−^^1^ decreases in intensity, and the broadband between 2400 and 3682 cm^−^^1^ also shows a decrease, indicating a reduction in encapsulated water. These transformations highlight the high affinity of DEZ for amide groups and encapsulated water, leading to structural modifications and the incorporation of zinc‐related species in the chitosan matrix, revealing the diversity of ALD processes.

**TABLE 5:** ATR‐FTIR spectra of Chitosan film and its DEZ functionalized films; samples VMP, MPI and PEALD.

CS film (70% Chitosan)	Sample VPM	Sample MPI	Sample PEALD	Assignment
—	601, 618, 725	602, 619, 725	601, 619, 668, 725	Zinc hydroxide and related species [116, 117]
1024.2 1064.3	991.0, 1022.9, 1066.0	993.0, 1023.8, 1061.4	991.0, 1023.8, 1063.4	1030‐1080 cm^−^^1^: C‐O‐C and C‐O‐ stretching (glycosidic linkages and ‐CH_2_‐OH group)
1375.2	1377.7	1375.4	1376.5	1300‐1380 cm^−^^1^: C‐N stretching vibrations
1407.2	1410.5	1410.4	1408.1	CH_2_ bending
1459.2	1444.9	1442.4	1436.7	‐C‐N‐H
1539.9	1535.9	1546.5	1543.5	1500‐1550 cm^−^^1^: N‐H stretching (amine II band from chitosan)/Amide II band (N‐H bending coupled with C‐N stretching)
1575.3	1565.9	1584.4	1576.3	1550‐1650 cm^−^^1^: Amide I band (C = O stretching from chitin)
1635.4	1633.5	1636.1	1631.5	
2790.7	2749.7	2749.3		2850–2920 cm^−^^1^: C‐H stretching (associated with methylene groups)
2860.8	2895.5	2852.7	2861.2	
2918.7	2951.9	2919.9	2921.3	
3135.9	3147.0	3198.4	3201.7	3000‐3500 cm^−^^1^: O‐H and N‐H stretching (overlapping region),
3272.7	3241.8	3258.3	3256.8	
3400	3391.3	3384.7	3402.4	
3514.9	3544.5	3509.3	3516.7	‐OH from water

Based on the results from the ATR‐FTIR and XPS analyses of the DEZ‐VPM, DEZ‐MPI, and DEZ‐PEALD samples, each process presents unique advantages. The DEZ‐VPM method, which involves a straightforward pulsing and purging sequence, offers simplicity and efficiency. It results in structural modifications, such as the reduction of amide group peaks, indicating effective interaction between DEZ and the chitosan matrix. The DEZ‐MPI process, involving the pulsing of DEZ and water, enhances interaction with the chitosan matrix, leading to increased protonated amine peaks and significant reductions in encapsulated water. This method demonstrates precise control of metalation with a lower zinc concentration compared to VPM. Lastly, the DEZ‐PEALD process, using O_2_ plasma‐enhanced ALD, improves the crystallinity and optoelectronic properties of ZnO films, effectively reduces defects, and modifies surface states for better performance in optoelectronic devices. The selective removal of absorbed water and the incorporation of zinc‐related species result in significant structural modifications, making PEALD a highly effective method for achieving high‐quality ZnO films in the chitosan matrix.

### 3.4 Surface Topography and Wetting Characteristics

The surface topography of the ZnO‐functionalized chitosan scaffolds, synthesized via VPM, MPI, and PEALD, was characterized using Atomic Force Microscopy (AFM), as shown in Figure 6. To assess the mesoscopic deposition behavior, 50 µm × 50 µm scans were performed, followed by 5 µm × 5 µm scans for high‐resolution nanoscale quantification. The root mean square roughness (Rq) served as the primary statistical metric to evaluate surface variations across the different processing regimes. The native chitosan surface (Figure 6a) presents a heterogeneous topography (Rq = 75.5 nm) likely influenced by residual chitin and acetic acid, which introduce an uneven distribution of nucleophilic sites. Interestingly, the CS–VPM sample (Figure 6b) shows a dramatic planarization effect (Rq = 5.6 nm), where the absence of a second precursor allows DEZ to infiltrate and smooth the surface defects. In sharp contrast, both the MPI and PEALD processes favor the formation of surface nucleation, though with vastly different topographical outcomes. For the CS–MPI sample (Figure 6c), the sequential pulsing of DEZ and H_2_O leads to the highest observed roughness (Rq = 123.9 nm), a direct consequence of aggressive in situ ZnO nucleation and the growth of large inorganic aggregates. Conversely, the CS–PEALD sample (Figure 6d) exhibits a more refined, textured morphology with a significantly lower roughness (Rq = 36.9 nm) than both the MPI and pristine CS surfaces. This suggests that the O_2_ plasma treatment facilitates a smoothing effect on the initial polymer substrate, likely through the simultaneous micro‐etching of prominent surface asperities and the promotion of a higher nucleation density. By creating a more uniform distribution of reactive sites, the plasma‐enhanced process favors the growth of smaller, more coalesced ZnO clusters. These observations highlight that while the introduction of a second reactant shifts the growth mechanism toward surface structuring, the use of O_2_ plasma specifically modulates this nucleation to produce a more homogenized and nanostructured hybrid interface.

**FIGURE 7:**
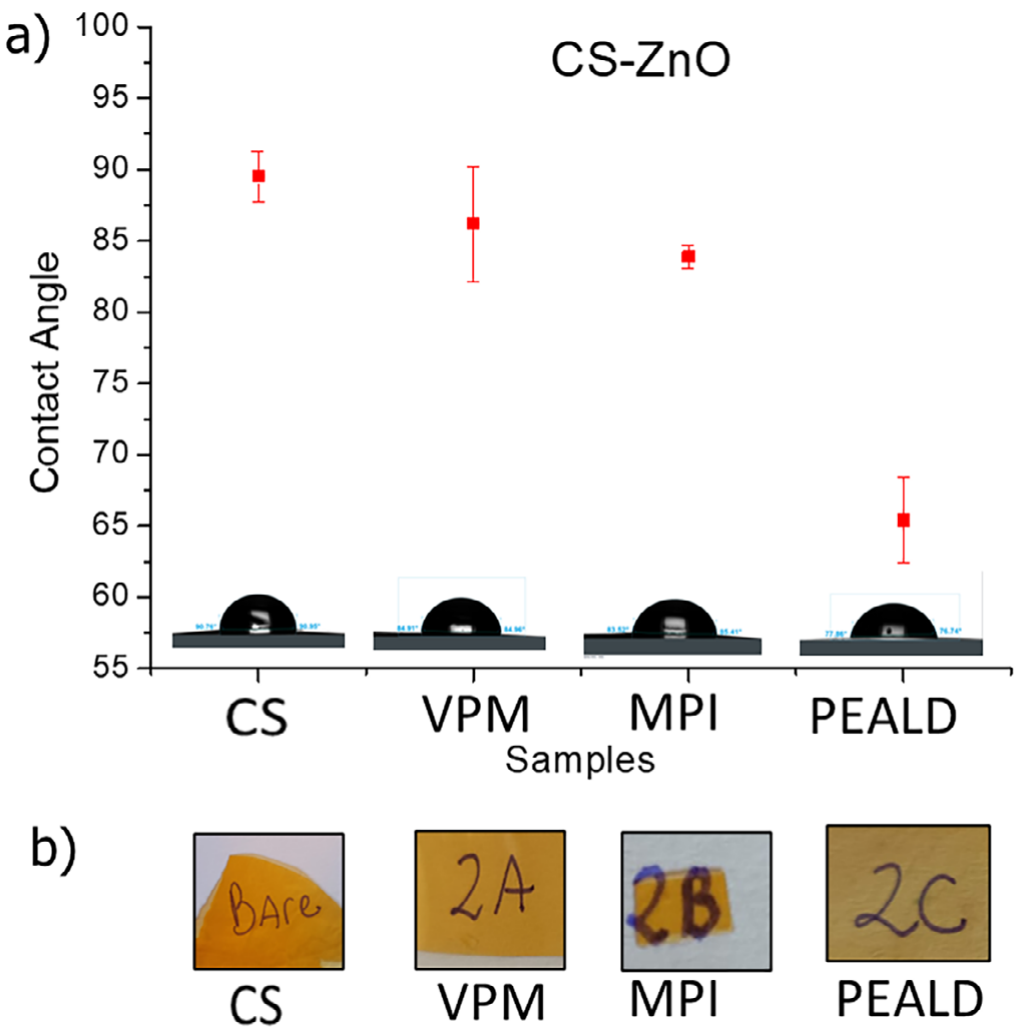
(a) Contact angle measurements of chitosan (CS) and its ZnO‐modified counterparts obtained using the ALD methods VPM, MPI and PEALD. Data represent mean ± SD (n = 5–10). (b) Photographs of the corresponding sample surfaces (CS, VPM, MPI, PEALD) taken with a 48‐megapixel camera.

To quantify the energetic favorability of water interaction with the CS–ZnO surfaces, the apparent solid–liquid interfacial energy (−ΔG_SL_) was derived using a modified Young–Dupré equation 1 [99], integrating both the water contact angle (WCA) data (Figure 7a) and AFM roughness parameters, as summarized in Table 6. While the pristine CS film presented a near‐hydrophobic contact angle of 89.5°, all modified scaffolds underwent a hydrophilic transition. Notably, the PEALD sample exhibited the most significant shift, reaching 65.4° and yielding the highest interfacial energy (0.102 J/m^2^). This lower contact angle indicates better swelling properties for the PEALD regime, as the increased surface wettability facilitates the infiltration of aqueous media into the chitosan matrix. This confirms that the plasma‐enhanced process creates the most thermodynamically receptive surface, surpassing the wetting performance of the VPM and MPI regimes.

**TABLE 6:** Non‐intrinsic solid–liquid interfacial energy (−ΔG_SL_) derived from contact angle measurements and AFM roughness parameters using water as probe liquid.

Sample	θ	Cos (θrad)	Roughness Rq (nm)	Roughness Factor (r ≈ 1 + Rq/1000)	−ΔG_SL (J/m2)
CS‐Zn VPM	86.2	0.07	5.6	1.0	0.078
CS‐Zn MPI	83.9	0.11	123.9	1.1	0.080
CS‐Zn PEALD	65.4	0.42	36.9	1.0	0.102
CS	89.5	0.01	75.5	1.1	0.073

This enhanced wettability in PEALD is attributed to the synergistic effect of O_2_ plasma activation and ZnO nucleation dynamics. The plasma treatment promotes the dehydration of the CS film and the efficient removal of surface carbonate species—noted by the comparative solubility products of ZnO (Ksp: 2.2*10^−17^) and CaCO_3_ (Ksp: 3.3*10^−9^) [117]. This surface refinement increases the accessibility of polar groups upon subsequent rehydration of the polymer matrix. In contrast, VPM and MPI samples showed a less pronounced decrease in contact angle. In these processes, the predominant species is likely zinc hydroxide (Zn(OH)_2_: 1.7×10^−^^1^ ^7^ Ksp) [119], which stems from the interaction between anchored metal ions and the residual encapsulated water within the CS matrix.

The superior affinity for water in the CS‐ZnO‐PEALD sample is particularly noteworthy given that its roughness (36.9 nm) is lower than the pristine CS (Table 6), suggesting that surface chemistry—specifically ZnO enrichment and plasma‐induced rehydration—dominates the wetting behavior over simple topographic effects. Conversely, CS‐ZnO‐MPI presented a moderate ΔG_SL_ (0.080 J/m^2^), where the influence of the highest roughness (123.9 nm) is tempered by the mixed ZnO/Zn(OH)_2_ chemistry. The VPM sample yielded the lowest energy (0.078 J/m^2^), likely due to dense ZnO nucleation in the absence of external oxidants, which may sterically suppress the accessibility of the polymer's polar functional groups.

These findings confirm that the interfacial energy of these hybrid scaffolds is not governed solely by topography, but by a complex interplay of deposition chemistry, Zn‐species distribution, and the hydration state of the chitosan backbone. This behavior is further modulated by the synergistic interactions between N–C = O, ‐NH_2_, encapsulated water, and anchored metal ions within the biopolymer matrix, which collectively define the reactivity and biological potential of the modified surface.

The macroscopic appearance of the membranes (Figure 7b) shows no visible differences among the samples, as all films present a similar translucent brown coloration. This confirms that the modifications introduced by VPM, MPI and PEALD occur at the nanoscale, rather than producing detectable changes in bulk appearance.

### 3.5 Bacterial Growth Inhibition Test and Cell Proliferation

The successful integration of advanced materials into biological systems hinges on achieving superior biocompatibility. A critical challenge lies in engineering high‐fidelity biotic/abiotic interfaces that minimize tissue damage, reduce peri‐implant scarring, and enhance long‐term operational stability [14]. In this context, antibacterial activity is paramount; preventing infections from multi‐resistant microbes is essential to avoid inflammatory cascades that compromise implant efficacy.

**FIGURE 8:**
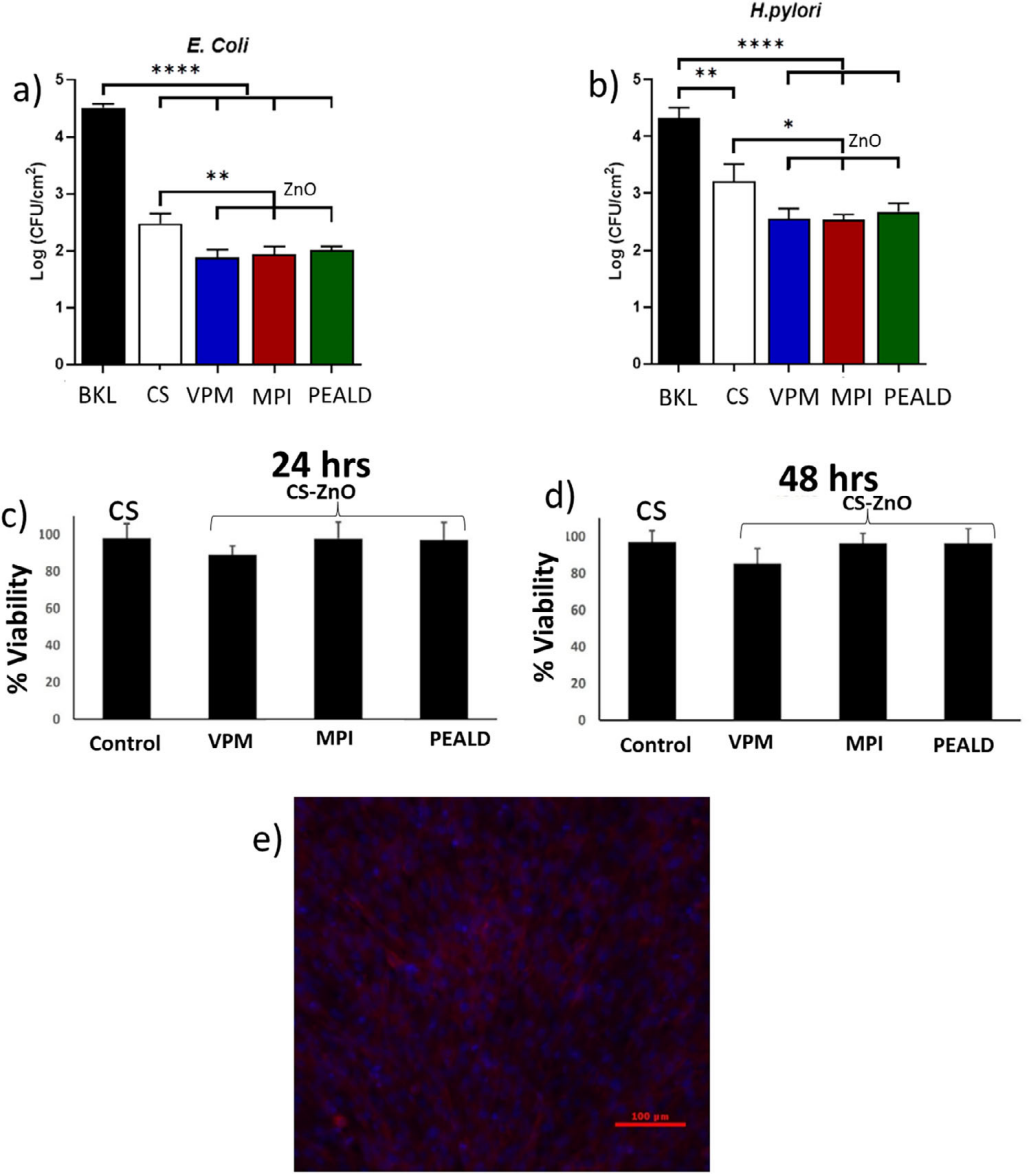
Count of viable colonies of (a) *Escherichia coli* and (b) *Helicobacter pylori* exposed over the surface of modified membranes. Over 1cm^2^ was deposited 100 µL of a solution containing 3 × 10^6^ CFU/mL and maintained for 24 h at 37°C. Later, bacteria were recovered and diluted to deposit on a lysogeny broth agar plate to final count formed colonies. (n = 3, **p < 0.01; ***p < 0.001; ****p < 0.0001). C2C12 cell biocompatibility test: cell viability (WST‐1 assay) data represent mean ± SD from triplicate measurements (n = 3) on Chitosan membranes at 24 h (c) and 48 h (d). (e) Epifluorescence micrograph of C2C12 cells cultured on the CS–ZnO PEALD sample.

The antibacterial performance of the modified scaffolds was evaluated by quantifying colony‐forming units (CFU) recovered from defined surface areas (Figure 8a, b). As expected, pristine chitosan (CS) exhibits inherent antibacterial properties due to its polycationic structure, which interacts with negatively charged bacterial cell wall components—such as phospholipids and lipopolysaccharides—leading to membrane permeabilization and cell death [120, 121]. Notably, all three ALD‐modified CS membranes demonstrated significantly enhanced reduction of both *E. coli* and *H. pylori* CFUs compared to unmodified CS. The VPM process showed superior inhibition against *E. coli*, while MPI exhibited enhanced activity against *H. pylori*. This divergence underscores the influence of the specific Zn‐metalation mechanism: the VPM process anchors Zn atoms within the CS matrix via *in‐situ* nucleation utilizing encapsulated water, highlighting the role of endogenous hydration in defining antiseptic properties. Interestingly, the superior performance of VPM and MPI over the ZnO‐coated PEALD samples suggests that a hybrid system incorporating Zn–CS coordinated species offers more effective antiseptic synergy than a simple surface coating. Furthermore, this atomic‐scale functionalization leverages ZnO's antibacterial potential while minimizing bulk toxicity concerns. The interplay between cells and material surfaces is governed by an intricate balance of physicochemical properties that dictate adhesion, proliferation, and migration [122]. This interaction is mediated by the adsorption of serum proteins (e.g., fibronectin and vitronectin) that act as bridges between the material surface and cellular integrins [123, 124]. As shown in Figure 8c–e, our Zn‐functionalized films provide an optimized environment for C2C12 myoblast proliferation. This positive response stems from the anchoring of Zn within the CS matrix (poly‐b‐1,4‐linked D‐glucosamine and N‐acetyl‐D‐glucosamine), which increases the accessibility of polar oxygen and nitrogen groups. Consistent with our interfacial energy analysis, the PEALD samples exhibited higher swelling ability and improved wettability (0.102 J/m^2^), which directly correlates with the enhanced cell viability observed. While excessively hydrophobic surfaces impede initial adhesion, the controlled atomic‐scale deposition in the VPM, MPI, and PEALD regimes yields a finely tuned hydrophobic/hydrophilic balance. This equilibrium promotes robust protein adsorption and stable cellular anchoring. Figure 8e confirms this through epifluorescence microscopy, showing a confluent C2C12 monolayer across the CS‐ZnO PEALD surface with no evidence of cytotoxicity. The enhanced conductivity and superior hydration state (swelling) of the PEALD interface likely promote intercellular signalling and connectivity, fulfilling the essential criteria for next‐generation intelligent medical implants and tissue regeneration [125, 126].

### 3.6 In Vivo Macroscopic Evaluation and Tissue Integration

The robust cellular proliferation and balanced antiseptic properties observed in vitro provided the fundamental rationale for evaluating the clinical potential of these hybrid interfaces in vivo. Building upon the superior biocompatibility, optimized hydration (swelling), and high interfacial energy (0.102 J/m^2^) demonstrated by the plasma‐modified surfaces, the CS‐ZnO PEALD scaffold was identified as the lead candidate for animal studies. Following the international Animal Welfare Code and the 3Rs principle (Replacement, Reduction, and Refinement), the in vivo assessment was prioritized for the PEALD regime to minimize animal usage while focusing on the most promising functionalization strategy. Consequently, we proceeded with a subcutaneous implantation model comparing pristine CS and CS‐ZnO PEALD to assess tissue integration and real‐time biological response.

**FIGURE 9:**
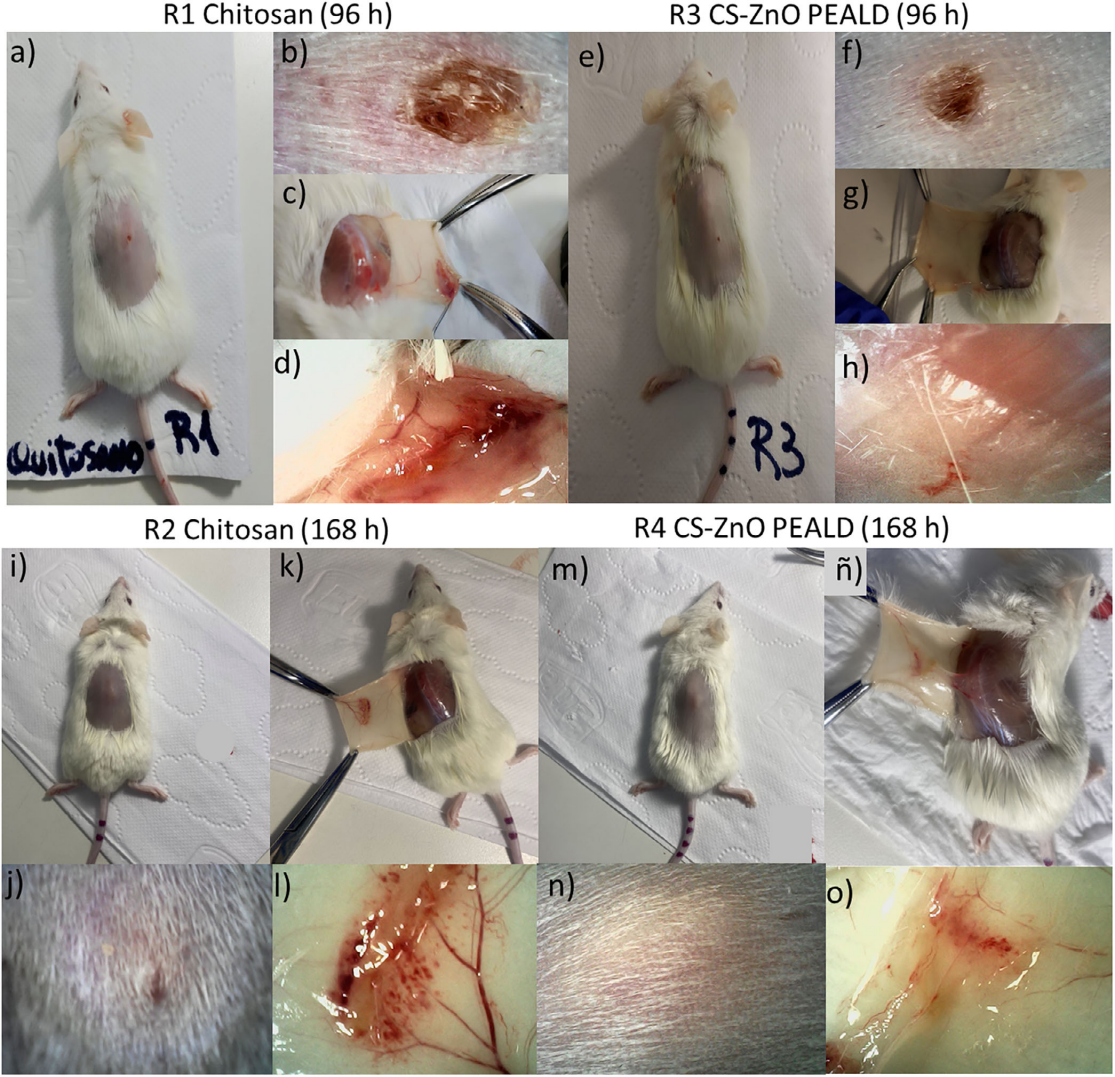
Macroscopic evaluation of the in vivo response and tissue integration. (a–h) Acute phase at 96 h post‐implantation: (a–d) Chitosan (CS) control group (R1) and (e–h) CS–ZnO PEALD group (R3). (i–o) Resolution phase at 168 h: (i–l) CS control group (R2) and (m–o) CS–ZnO PEALD group (R4). The images demonstrate the stable positioning of the polymeric scaffolds achieved through a minimally invasive CORE‐type approach without surgical sutures. Close‐up views in panels (d, h, l, o) highlight the healthy dermal‐matrix interface, showing early integration with no clinical signs of exacerbated inflammation, suppuration, or tissue necrosis in any of the experimental groups.

The macroscopic evaluation confirmed that the nanometric ZnO coating via PEALD does not compromise the high biocompatibility of the chitosan matrix (Figure 9). The use of a CORE‐type minimally invasive surgical approach ensured the stable integration of both Chitosan (CS) and CS–ZnO PEALD scaffolds within the subcutaneous pocket without the need for sutures. During the initial phase (96 h), the harvested tissues showed a clean interface with the host tissue. Both the control (Figure 9a–d) and the ZnO‐modified scaffolds (Figure 9e–h) were firmly positioned, exhibiting initial adhesion and a healthy surrounding dermis. No signs of acute rejection or adverse reactions were detected at this stage. By 168 h post‐implantation, the integration had progressed significantly for both materials (Figure 9i–o). Macroscopic inspection revealed that the matrices were well‐incorporated into the subcutaneous tissue, maintaining their structural integrity. Notably, while the pure Chitosan control showed persistent small hematomas or blood clusters at the interface (Figure 9j, l), the CS–ZnO PEALD group exhibited a visibly cleaner integration with a reduction in blood accumulation (Figure 9n, o). This suggests a more refined healing process and a faster resolution of the initial surgical trauma in the presence of the ZnO coating. These high‐magnification views (panel o) revealed a localized and organized vascular response, providing a solid macroscopic basis for the angiogenic and systemic cytokine analyses presented in the subsequent sections.

**FIGURE 10:**
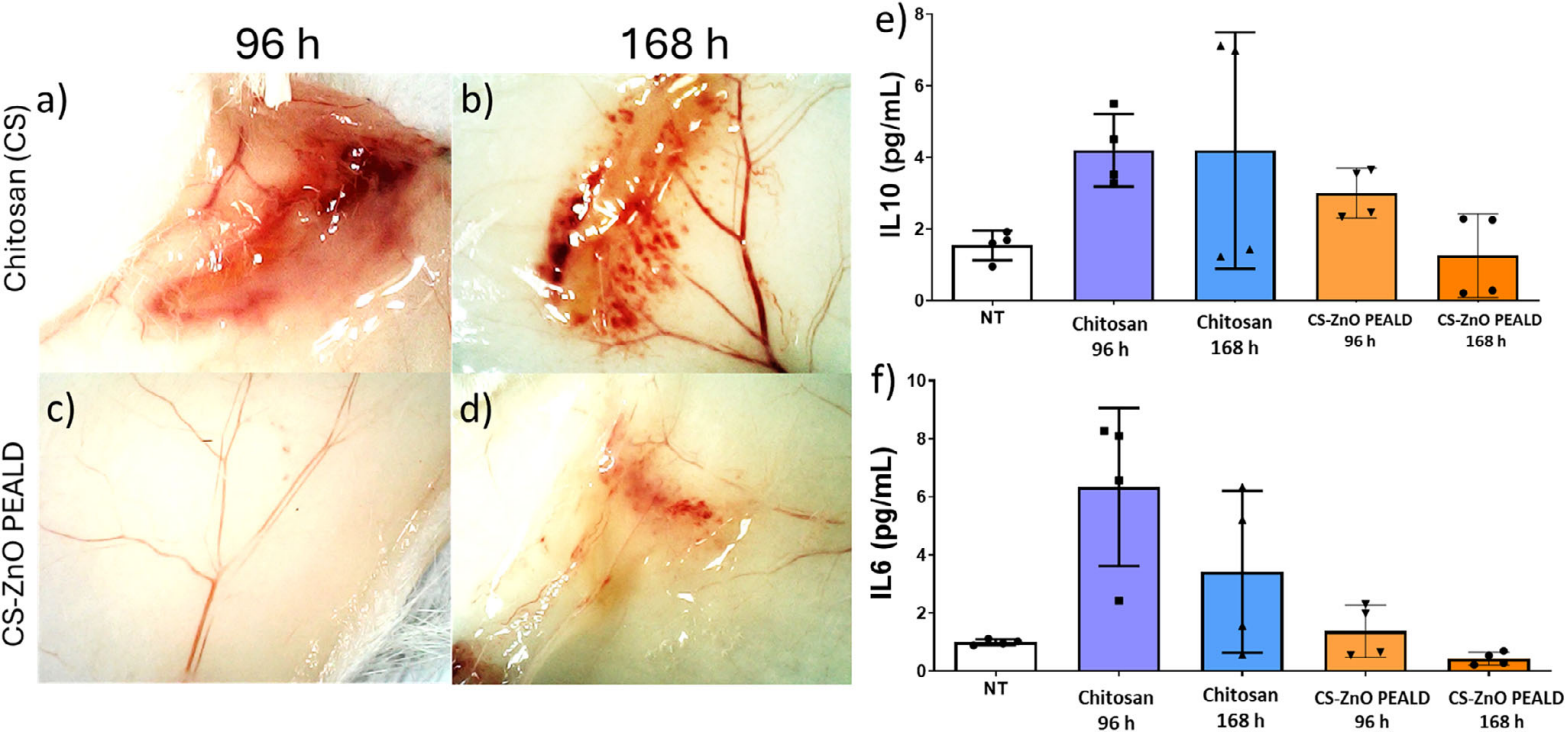
Local angiogenic evaluation and systemic cytokine modulation. (a–d) High‐magnification macroscopic views of the subcutaneous interface at 96 and 168 h. A localized vascular response is observed in both groups; however, the CS–ZnO PEALD group (c, d) exhibits a more organized vascular distribution with a notable reduction in residual hematomas compared to the pure Chitosan (CS) control (a, b) at 96 h and 168 h, respectively. (e, f) Quantitative analysis of circulating cytokines in murine plasma: (e) IL‐10 and (f) IL‐6 concentrations (pg/mL). The data reveal that ZnO‐coated scaffolds promote a balanced immune environment, characterized by stable anti‐inflammatory IL‐10 levels and significantly lower pro‐inflammatory IL‐6 expression compared to the CS control at both time points. Data represent mean ±SD (n = 2 per group). NT: Non‐treated control.

To further investigate the biological impact of the nanometric ZnO coating, the local vascular response was correlated with the systemic inflammatory profile (Figure 10). High‐magnification analysis of the harvested tissues revealed active angiogenesis at the implantation site. Notably, while the pure Chitosan control exhibited persistent blood clusters and more pronounced hematomas at 96 and 168 h (Figure 10a, b), the CS–ZnO PEALD scaffolds showed a cleaner integration interface with a more refined and organized microvascular network (Figure 10c, d). This improved local resolution was supported by the systemic cytokine analysis. The pro‐inflammatory marker IL‐6 was markedly lower in the CS–ZnO PEALD group at both 96 h (1.4 ± 1.2 pg/mL) and 168 h (0.5 ± 0.3 pg/mL) compared to the pure Chitosan group, which showed higher and more variable expression (Figure 10f). Furthermore, the anti‐inflammatory cytokine IL‐10 remained consistently expressed in the presence of the ZnO‐modified scaffolds, promoting a balanced immune environment (Figure 10e). The combination of reduced local haemorrhage, significantly lower systemic IL‐6 levels, and sustained IL‐10 expression suggests that the PEALD‐derived ZnO coating successfully modulates the early inflammatory phase, accelerating the transition toward a pro‐regenerative and angiogenic environment. These findings validate the use of atomic layer deposition as a biocompatible strategy to enhance the performance of chitosan‐based surgical matrices.

The integration of the biochemical data from Table 7 with the macroscopic observations in Figures 9 and 10 reveals a significant advantage of the PEALD‐modified scaffolds. While Chitosan is a well‐known biocompatible polymer, our results indicate that the addition of atomic ZnO layers further refines the host's immune response. The marked reduction in IL‐6 levels in the CS–ZnO PEALD group—reaching near‐basal concentrations by 168 h (0.5 ±0.3 pg/mL)—suggests that the ZnO coating may act as a modulator of the initial inflammatory surge. This is complemented by the presence of IL‐10 in both groups, which reflects a physiological regulatory response aimed at maintaining immune homeostasis.

**TABLE 7:** Systemic inflammatory cytokine profile in murine plasma at 96 and 168 h post‐implantation of Chitosan (CS) and CS–ZnO PEALD scaffolds.

Analyte Name	Time Point	Chitosan (pg/mL)	S.D	Chitosan‐ZnO PEALD (pg/mL)	S.D
IL‐10	96 h	4.2	1.1	3.0	0.9
	168 h	4.2	4.0	1.2	1.5
IL‐17A	96 h	0.0	0.0	0.0	0.0
	168 h	0.0	0.0	0.0	0.0
TNF	96 h	0.0	0.0	0.0	0.0
	168 h	0.5	0.7	0.0	0.0
IFN‐γ	96 h	0.0	0.0	0.0	0.0
	168 h	0.0	0.0	0.0	0.0
IL‐6	96 h	5.3	4.1	1.4	1.2
	168 h	3.4	4.1	0.5	0.3
IL‐4	96 h	0.0	0.0	0.0	0.0
	168 h	0.0	0.0	0.0	0.0
IL‐2	96 h	0.7	0.4	0.5	0.1
	168 h	0.8	0.3	0.1	0.0

This systemic profile correlates directly with the local tissue resolution: the ‘cleaner’ interface and the organized microvasculature observed in the ZnO‐treated mice (Figure 9d) are likely a result of this attenuated inflammatory signalling. In contrast, the higher and more persistent IL‐6 levels in the pure Chitosan group coincide with the presence of small residual hematomas at 168 h (Figure 9l), indicating a slightly more prolonged recovery phase. Furthermore, the low and stable levels of MCP‐1 (0.8 ±0.7 pg/mL at 168 h) and IL‐2 (0.1 ±0.0 pg/mL at 168 h) in the CS–ZnO PEALD group indicate that the material does not trigger significant monocyte recruitment or uncontrolled T‐cell proliferation. The absence of IL‐4 and IL‐17A across all time points also confirms that the scaffolds do not activate Th2‐allergic or Th17‐mediated chronic inflammatory pathways. Finally, the lack of TNF and IFN‐γ throughout the study further confirms that the release of Zn^2+^ ions from the ALD coating occurs within a safe therapeutic window, promoting angiogenesis without triggering systemic toxicity. These findings position CS–ZnO PEALD as a superior candidate for surgical applications where rapid integration and minimal inflammation are required. Additional high‐resolution evidence of the tissue interface—specifically highlighting the reduction of haemorrhagic clusters in the ZnO‐modified group—is provided in the Electronic Supplementary Information (ESI), Figures S7–S9.

## 4 Summary

The study underscores the critical role of both endogenous and exogenous water, along with Zn‐OH, in shaping the structure and properties of the resulting materials. Zn infiltration significantly influences the intra‐ and intermolecular interactions within CS networks, particularly the hydrogen bonds involving H_2_N–/ N–(C = O)‐ and HO groups. The availability of free hard H_2_N‐, ‐OH/H_2_O centres within CS dictates the extent of these interactions and, consequently, the properties of the Zn‐CS composites.

Structural and encapsulated water in CS facilitates the hydrolysis of diethylzinc to Zn‐hydroxide, partially disrupting the polymer network. This disruption increases the concentration of available ‐NH_2_ and ‐OH groups for coordinating with additional zinc ions, as observed in the VPM process.

X‐ray diffraction analysis quantifies this structural metamorphosis; the consumption of internal water by DEZ in the VPM regime causes a significant contraction of the hydrated phase crystallite size from 7.4 nm to 4.6 nm. In contrast, the introduction of a second precursor in MPI and PEALD leads to a more balanced reorganization of the crystalline network, increasing the regular‐to‐hydrated area ratio from 1.7 to 2.1. In the MPI process, the high density of nucleation sites prevents large crystal growth—reducing crystallite sizes to 2.8 nm—which provides the physical basis for the observed topographical shifts.

SEM and AFM characterization reveal that this interaction can be used to precisely tune surface topography, ranging from the planarization effect observed in VPM (Rq = 5.6 nm) to the high‐surface‐area nucleation found in MPI (Rq = 123.9 nm). While the MPI process results in lower Zn deposition than VPM, PEALD—utilizing pulsed oxygen plasma—produces both Zn‐hydroxide and ZnO due to complete CS dehydration.

These findings demonstrate that tuneable Lewis's acid‐base interactions primarily govern the physicochemical properties and potential applications of these Zn‐CS composites. This tunability allows for tailoring material properties by simply selecting the appropriate metalorganic precursor.

Four key criteria were employed to evaluate the suitability of these materials for intelligent implant design:

1. Wettability and Interfacial Energy: PEALD samples exhibited a significant hydrophilic transition and the highest interfacial energy (0.102 J/m^2^). This lower contact angle indicates better swelling properties for the PEALD regime, as the increased surface energy facilitates the infiltration and diffusion of aqueous media into the hydrophilic chitosan matrix. This indicates a thermodynamically receptive surface that promotes superior biological interaction compared to the near‐hydrophobic pristine CS.
2. Semiconductor Properties: Diffuse reflectance spectra revealed the semiconductor character of ZnO‐CS films, with MPI samples displaying the most pronounced behavior.
3. Antiseptic Activity: VPM samples demonstrated superior antiseptic properties against *E. coli*, while MPI samples were more effective against *H. pylori*. This difference highlights the impact of Zn‐metalation methods on the CS network and the importance of Zn bond‐specific functionalization and ZnO‐CS synergy.
4. Biocompatibility and In Vivo Integration: While all modified films supported C2C12 cell viability, in vivo subcutaneous implantation confirmed that CS‐ZnO PEALD scaffolds promote excellent tissue integration, active angiogenesis, and the absence of exacerbated inflammatory responses. This performance represents a critical improvement over pristine CS scaffolds.
5. These promising ALD methodologies hold significant potential for developing advanced biomaterials for various applications, including drug delivery, quantum sensing, biosensors for reactive species and metabolites, and next‐generation intelligent medical implants. They offer a versatile platform for tailoring material properties and advancing the fields of BIOMEMS, NEMS, and ZnO‐based devices.

## 5 Conclusions

This study demonstrates the successful Zn‐functionalization of chitosan films through three distinct ALD‐based regimes: VPM, MPI, and PEALD. Our findings highlight the crucial role of both endogenous and exogenous water in determining the final physicochemical and biological properties of the hybrid interface. XPS and XRD analyses confirm a controlled chemical and structural metamorphosis; while VPM selectively targets the hydrated chitosan phase—reducing its crystallite size from 7.4 to 4.6 nm—the introduction of a second precursor in MPI and PEALD optimizes the regular‐to‐hydrated area ratio to 2.1, reflecting a more organized crystalline network.

Morphological analysis confirms that surface topography can be precisely engineered, ranging from the planarization effect in VPM (Rq = 5.6 nm) to high‐surface‐area nucleation in MPI (Rq = 123.9 nm). A key milestone is the transition from the near‐hydrophobic nature of pristine CS to a highly hydrophilic state in PEALD samples, achieving the highest interfacial energy (0.102 J/m^2^). This lower contact angle indicates superior swelling properties, as the increased surface energy facilitates the infiltration of aqueous media into the chitosan matrix. This thermodynamic refinement directly correlates with the superior biological performance and antiseptic activity against *E. coli* and *H. pylori*.

Most significantly, in vivo subcutaneous implantation validated that CS‐ZnO PEALD scaffolds are superior to pristine CS, promoting active angiogenesis and exhibiting an immunomodulatory effect characterized by near‐basal pro‐inflammatory IL‐6 levels (0.5 pg/mL) and a balanced IL‐10 profile. These findings establish ALD‐functionalized chitosan as a premier candidate for next‐generation bio‐integrated electronics and intelligent medical devices. Future research will focus on leveraging this high biocompatibility and safety profile to develop advanced BCI probes and responsive drug delivery systems.

## Author Contributions

M.M, H.S., L.V., S.D., E.B, M.A. and V.C conceived and planned the synthesis and characterization of CS films; XRD, ATR‐FTIR, UV–visible spectra, AFM and contact angle properties. M.M., D.S., and A.D. designed and performed the functionalization of CS films using the VPM, MPI and O_2_ plasma processes implemented into ALD equipment. R.M performed XPS characterizations, R.M. and D.M. carried out the analysis of XPS analysis. M.K. contributed to the SEM and AFM characterization. Y.O. performed the C2C12 cell‐cultured and C2C12 Cell biocompatibility test on PA6. S.G., tested the antibacterial properties of the samples. M.M. and S.G. wrote the manuscript. All authors provided critical feedback and helped shape the research, analysis, and manuscript. S.G, L.L., and K.G. carried out the in vivo studies.

## Funding

This work was supported by National Fund for Scientific and Technological Development (FONDECY)/1171803 Chile, EPSRC (EP/V055232/1, EP/R008841/1), and FONDECYT 1240757 and ANID PIA / APOYO AFB230003

## Conflicts of Interest

The authors declare no conflicts of interest.

## Supporting information




**Supporting File**: adhm71329‐sup‐0001‐SuppMat.docx.

## Data Availability

The data that support the findings of this study are available from the corresponding author upon reasonable request.
