# Supplementary material for: Atomic Layer Deposition Processes: Versatile Platforms for Engineering ZnO‐Chitosan Biointerfaces
Source: Adv Healthc Mater. 2026 Jun 17;15(26):e71329. doi: 10.1002/adhm.71329 (PMC13356493; doi:10.1002/adhm.71329)
Supplement: Supplementary file 1 — Supporting File: adhm71329‐sup‐0001‐SuppMat.docx. [file ADHM-15-0-s001.docx]

**Atomic Layer Deposition Processes: Versatile Platforms for Engineering ZnO-Chitosan Biointerfaces**

Mabel Moreno^1,2*^, Anjana Devi^3-5**^, David Zanders^5^, Miryam Arredondo^5^, Davide Mariotti^7^, Ruairi McGlynn^8^, Sindy Devis^1^, Simón Guerrero^9^, Eglantina Benavente^10,11^, Matias Alegría^10^, Yusser Olguin^12-14^, Lorena Lobos-Gonzalez^15^,

Kevin Guzmán^15^, Elizabeth Rivas-Yañez^16^, Paula Solar^17^, Valentin Cepus^1,18^, Michael Krause^1^, Luis Velásquez^2^.

^1^Hochschule Merseburg − University of Applied Sciences, Fachbereich Ingenieur- und Naturwissenschaften, Eberhard-Leibnitz-Str. 2, Merseburg 06217, Germany

^2^Instituto de Investigación Interdisciplinar en Ciencias Biomédicas SEK, Facultad de Ciencias de la Salud, Universidad SEK, Santiago 8320000, Chile

^3^Leibniz Institute for Solid State and Materials Research (IFW), Helmholtzstr. 20, D-01069, Dresden

^4^Faculty of Chemistry and Food Chemistry Dresden University of Technology (TUD)

^5^Inorganic Materials Chemistry, Ruhr-University Bochum, 44780 Bochum, Germany.

*^6^Queen’s University Belfast, University Rd, Belfast, BT7 1NN, UK*

*^7^Department of Design, Manufacturing & Engineering Management, University of Strathclyde, Glasgow, UK*

*^8^Ulster University, 2-24 York Street, Belfast, BT15 1AP, UK*

*^9^Facultad de ciencias químicas y farmacéuticas, Universidad de chile, Carlos Lorca Tobar 964, Santiago, Chile.*

*^10^Departamento de Química, Facultad de Ciencias Naturales, Matemática y Medio Ambiente, Universidad Tecnológica Metropolitana, Santiago, Chile*

*^11^Programa Institucional de Fomento a la Investigación, Desarrollo e Innovación (PIDi), Universidad Tecnológica Metropolitana, Santiago, Chile*

*^12^Federico Santa María, Departamento de Química y Medio Ambiente, Universidad Técnica Federico Santa María, Avenida España 1680, Valparaíso, Chile.*

*^13^Centro científico y tecnológico de Valparaíso (CCTVal), Universidad Técnica Federico Santa María, Avenida España 1680, Valparaíso, Chile.*

*^14^Centro de Biotecnología, Universidad Técnica Federico Santa María, Avenida España 1680, Valparaíso, Chile.*

*^15^Investigación en Dinámica Tumoral Mamaria (DiTMa, Laboratorio de Comunicaciones Celulares, Centro de Vesículas Extracelulares Metabolismo y Cancer (CEMC), Núcleo Interdisciplinario de Biología y Genética (NiBG), Instituto de Ciencias Biomédicas (ICBM), Facultad de Medicina, Universidad de Chile). Santiago de Chile, Independencia 1027, Independencia.*

*^16^Facultad de Medicina, Universidad de Atacama, 153601, Copiapó, Chile*

*^17^Universidad Finis Terrae, Avenida Pedro de Valdivia 1509, Santiago Región Metropolitana, Chile.*

*^18^Polymer Service GmbH Merseburg, Geusaer Str. 81f, Merseburg 06217, Germany*

*Emails: (*) mabel.moreno@uisek.cl, (**)a.devi@ifw-dresden.de*


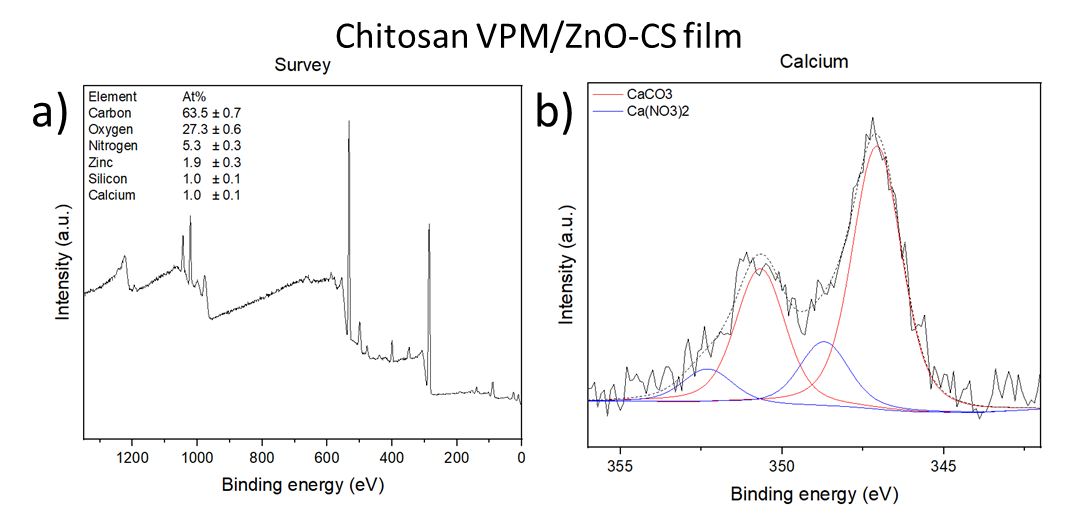


Figure S1. XPS survey (a) and Ca 2p (b) of sample VPM.


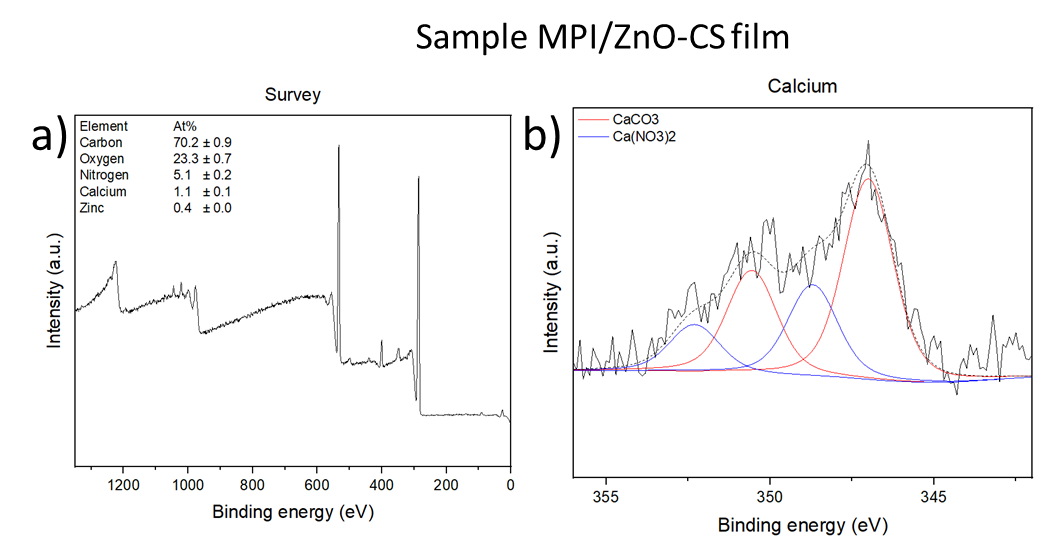


Figure S2. XPS survey (a) and Ca 2p (b) of sample MPI.


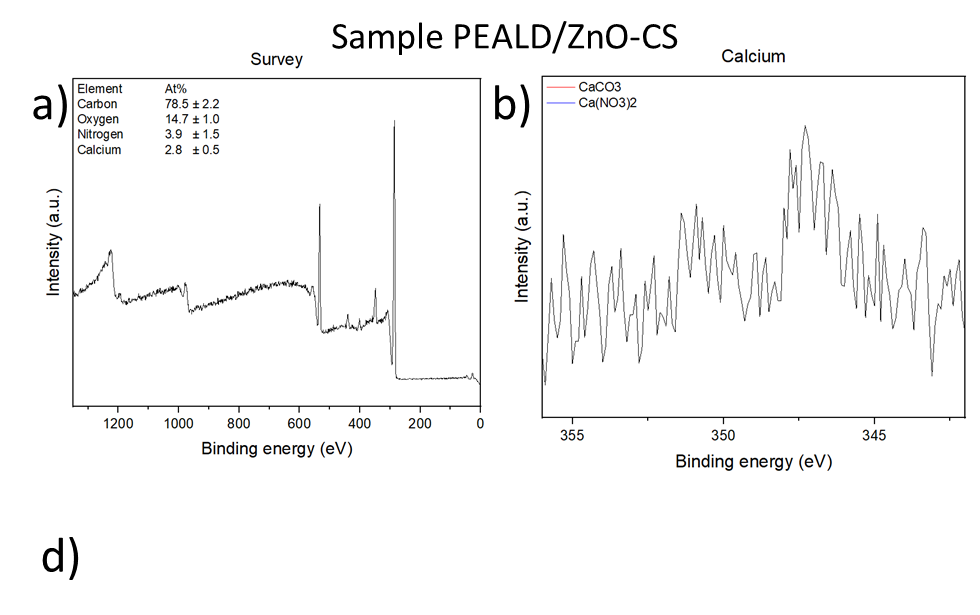


Figure S3. XPS survey (a) and Ca 2p (b) of sample PEALD.

Table S1. Crystallite Size and α/γ Ratio Calculation for CS film, samples VPM, MPI and PEALD

| Sample | 11.6° hydrated CS Crystallite size (nm) | 18.6° regular CS Crystallite size (nm) | 22.5° amorphous CS Crystallite size (nm) | Regular/Hydrated ratio (Area) | Regular/Amorphous ratio (Area) |
| --- | --- | --- | --- | --- | --- |
| CS | 7.4 | 2.24 | 2.7 | 8740/3351=2.6 | 8740/13771=0.64 |
| CS-ZnO VPM | 4.6 | 3 | 2.7 | 7806/4691=1.7 | 7806/1467=0.53 |
| CS-ZnO MPI | 2.8 | 1.83 | 3.3 | 8406/4029=2.1 | 8406/10063=0.84 |
| CS-ZnO PEALD | 4.8 | 2.3 | 2.4 | 9873/4733=2.1 | 9873/17279.=0.57 |


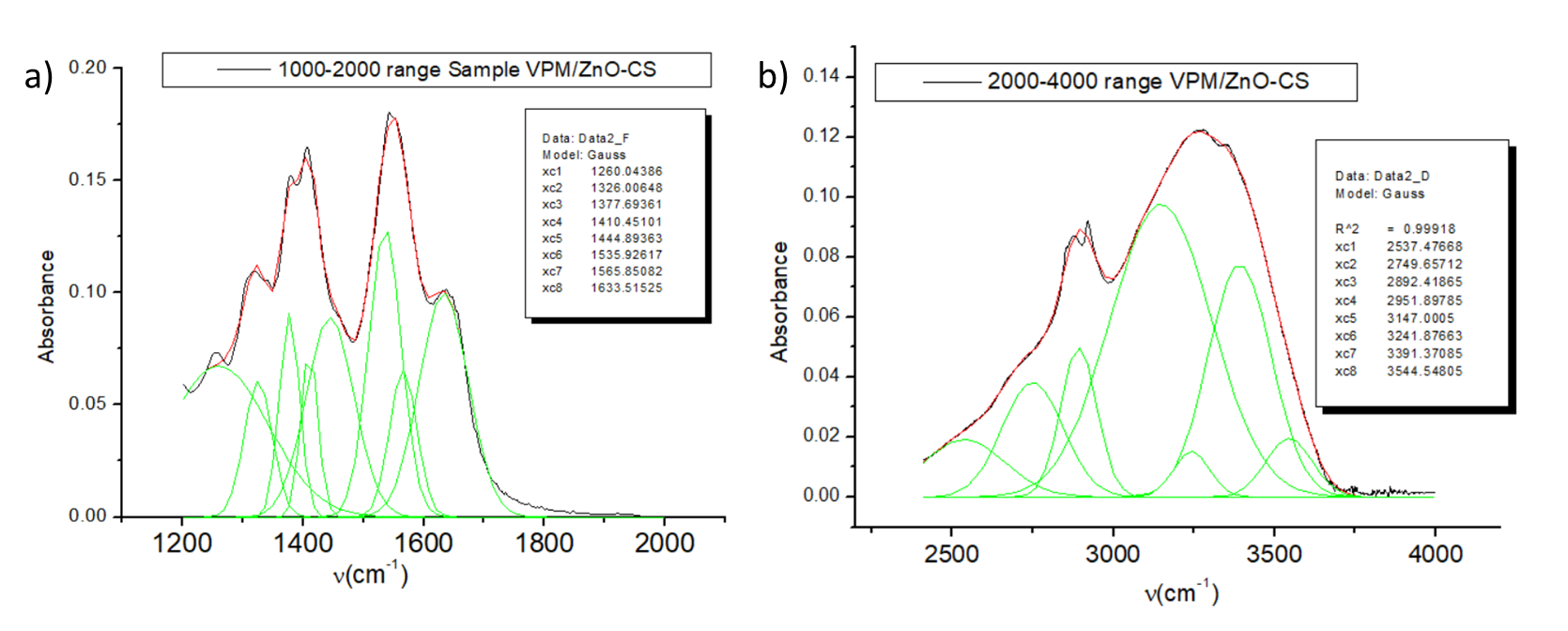


Figure S4. ATR-FTIR spectra of Sample VPM (a) 1000-2000 range, and (b) 2000-4000.


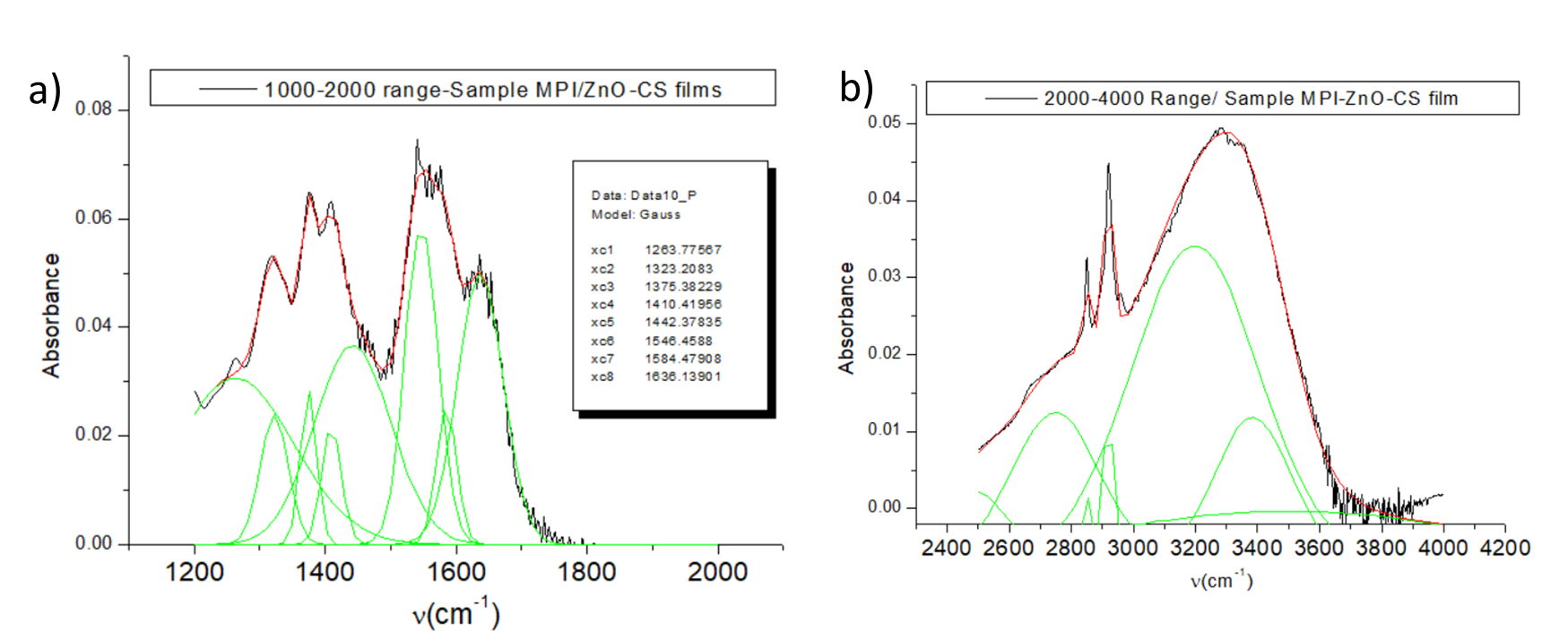


Figure S5. ATR-FTIR spectra of Sample MPI (a) 1000-2000 range, and (b) 2000-4000.


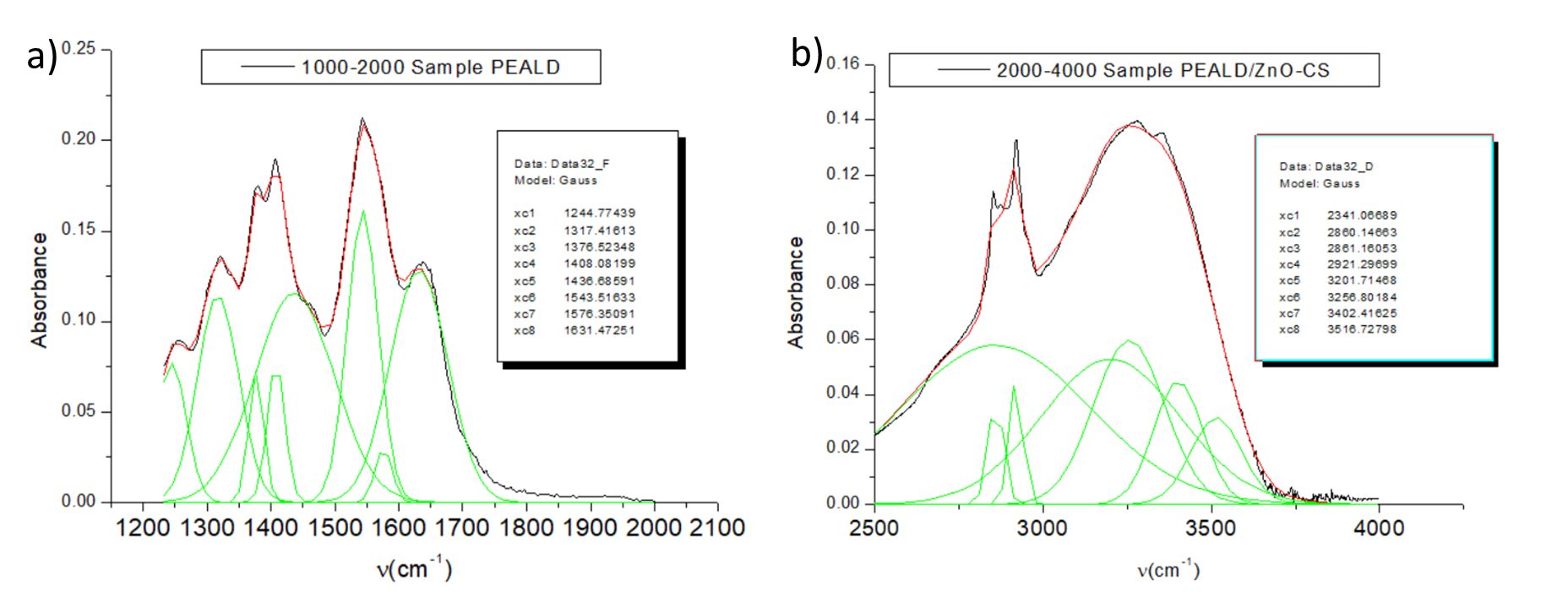


Figure S6. ATR-FTIR spectra of Sample PEALD (a) 1000-2000 range, and (b) 2000-4000.


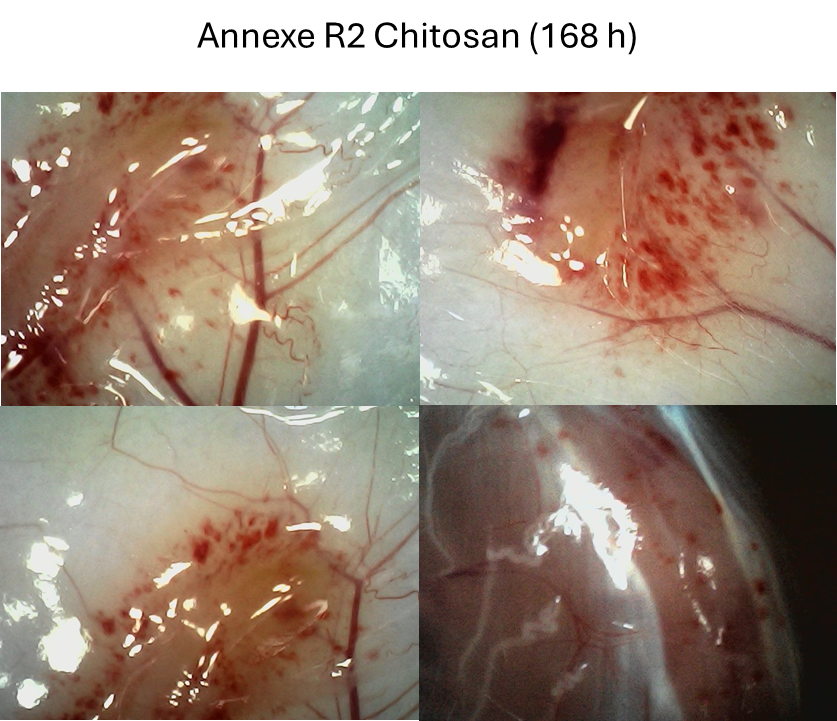


Figure S7. High-magnification macroscopic assessment of the Chitosan control interface (168 h). Representative images of the subcutaneous tissue interface in the Chitosan (R^2^) control group at 168 h post-implantation. The panels illustrate the morphological characteristics of the integration site, showing active microvascular recruitment and localised blood clusters typical of the inflammatory-to-proliferative transition phase in pure polymeric matrices. These detailed views highlight the vascular patterns and the physical adherence of the host tissue to the scaffold surface prior to histological processing.


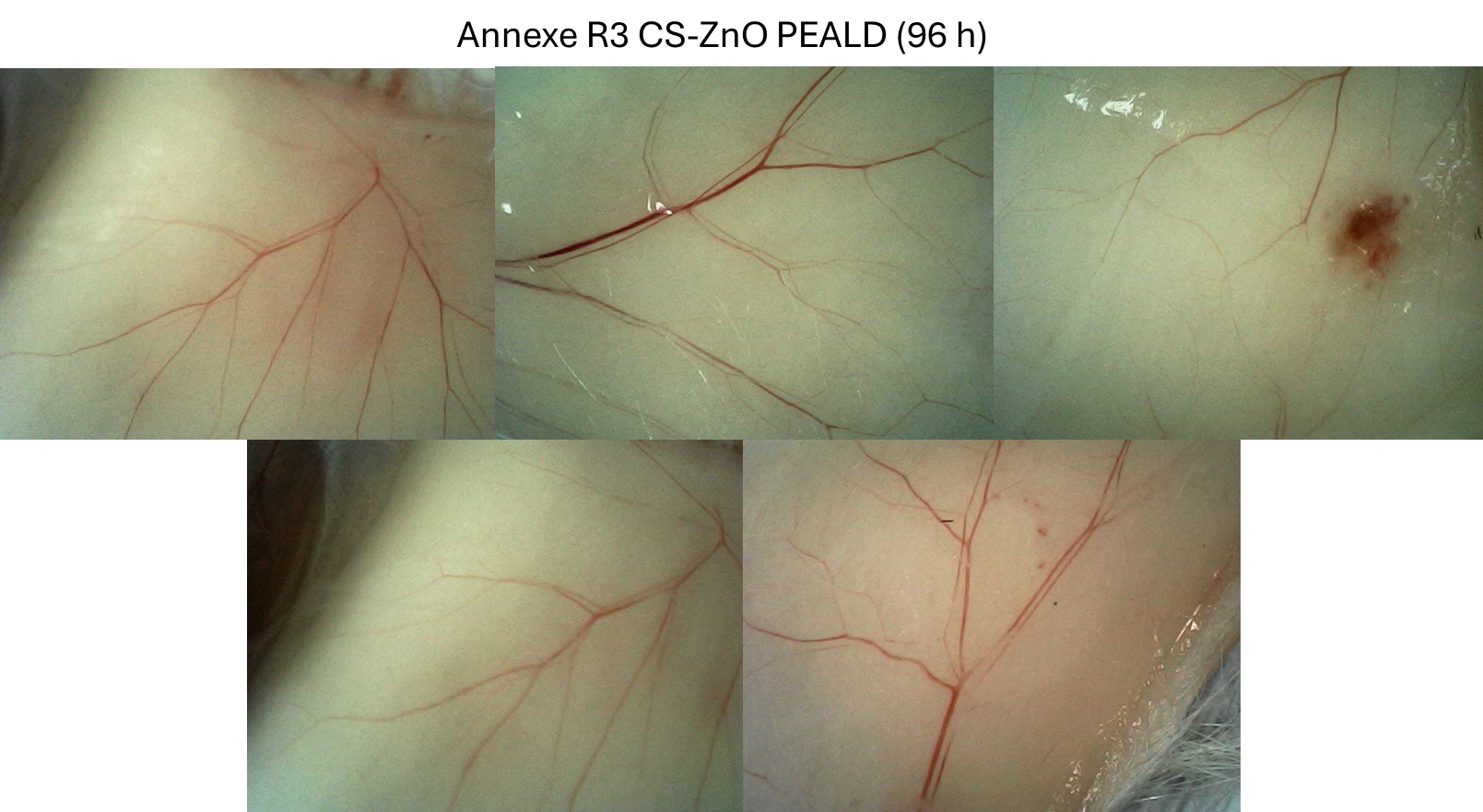


Figure S8. High-magnification macroscopic assessment of the CS–ZnO PEALD interface (96 h). Representative high-resolution images of the subcutaneous tissue interface for the CS–ZnO PEALD (R3) group at the acute phase (96 h post-implantation). The panels demonstrate a clean tissue-scaffold interface with an early, well-distributed microvascular network. Notably, even at this early stage, there is a lack of significant hemorrhagic clusters or exacerbated inflammatory signs at the integration site, supporting the high biocompatibility of the atomic ZnO layer. These detailed views provide visual evidence of the favourable host-material interaction before the full resolution observed at 168 h.


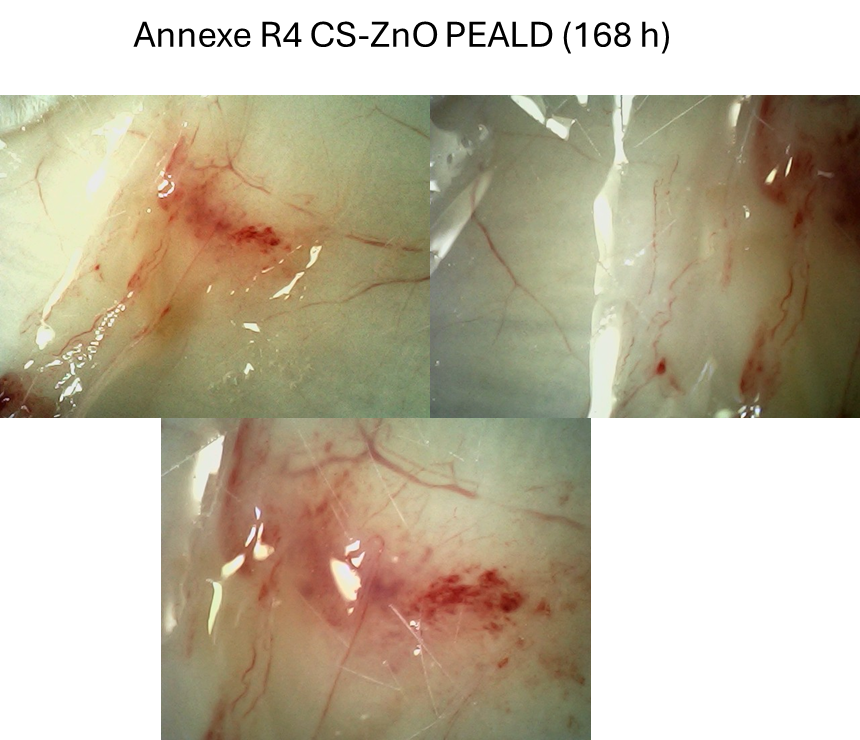


Figure S9. High-magnification macroscopic assessment of the CS–ZnO PEALD interface (168 h). Representative high-resolution images of the subcutaneous tissue interface for the CS–ZnO PEALD (R4) group at the resolution phase (168 h post-implantation). The panels reveal a highly integrated scaffold interface characterised by a reduction in vascular congestion and a significant absence of the large blood clusters observed in the pure chitosan group. The healthy appearance of the surrounding connective tissue and the presence of fine, organised microvasculature confirm that the atomic ZnO coating promotes a stable and biocompatible environment, facilitating the transition toward tissue remodelling.
